# Risk-Informed Complaint Management in Neurosurgery: Development and Temporal Validation of an Early-Warning Model for Dispute Escalation

**DOI:** 10.3390/healthcare14203360

**Published:** 2026-10-09

**Authors:** Mingming Zhang, Jiarong He, Yuquan Chen, Jian Cui, Yu Zhou

**Affiliations:** 1 Hunan Cerebrospinal Vascular Disease Diagnosis and Treatment Center, The Second Xiangya Hospital, Central South University, No. 139 Renmin Middle Road, Furong District, Changsha 410011, China; zhangmm@csu.edu.cn (M.Z.); 248201045@csu.edu.cn (J.H.); 248211141@csu.edu.cn (J.C.); 2 Department of Neurosurgery, The Second Xiangya Hospital of Central South University, Changsha 410011, China; 3 Faculty of Medicine, Nursing & Health Sciences, School of Public Health and Preventive Medicine, Monash University, Melbourne, VIC 3004, Australia; yuquan.chen@monash.edu; 4 Hunan Guangxiu HiTech Life Technology Co., Ltd., Changsha 410217, China; 5 National Engineering Research Center of Human Stem Cells, Changsha 410205, China

**Keywords:** neurosurgery, patient complaint, dispute escalation, early warning, risk stratification

## Abstract

**Background:** Neurosurgery is a high-risk surgical specialty with substantial clinical uncertainty, procedural complexity, and potential for severe adverse outcomes. Although timely risk stratification after an initial complaint may facilitate structured communication, senior review, and coordinated dispute management, practical tools for estimating escalation risk at the first complaint remain limited. **Methods:** We conducted a single-center retrospective study of neurosurgical complaint events recorded at the Second Xiangya Hospital from January 2016 to December 2025. Time zero was the first documented complaint date. The endpoint was a final institutional severity grade of 7–10, denoting hospital-level handling or beyond, versus grades 1–6. Candidate predictors were restricted to information available on or before time zero and were selected by univariable screening followed by multivariable logistic regression. The sociodemographic support-needs profile was excluded from the model to avoid stigmatization and was used in fairness analyses. **Results:** Among 975 eligible complaint events, 161 (16.5%) reached high-grade escalation. Four predictors were retained: ICU admission, invasive manipulation failure, a pre-complaint communication-context factor, and anger at the first complaint. The model achieved moderate discrimination and calibration in the apparent cohort (AUC, 0.781; PR-AUC, 0.417; Brier score, 0.115) and similar performance in the 2023–2025 temporal test cohort (AUC, 0.759 [95% CI, 0.687–0.826]; PR-AUC, 0.364; Brier score, 0.119). At a threshold of 0.20, the positive predictive value in the test cohort was 0.333. Exploratory risk strata separated high-grade escalation rates from 5.8% in the low-risk group to 16.6% and 37.3% in the intermediate- and high-risk groups. **Conclusions:** Routinely available clinical and communication indicators, recorded by the time of the first complaint, can support risk-informed complaint management in neurosurgery. Because two of every three high-risk flags did not escalate, the model should support senior review and early communication rather than serve as a stand-alone classifier; external validation and local recalibration are required before use elsewhere.

## 1. Introduction

Neurosurgery is widely regarded as a high-liability, high-acuity specialty [1,2]. It manages catastrophic neurological disease, invasive procedures, narrow margins for error, and outcomes that may involve death or permanent disability, and it requires repeated high-stakes communication with families under time pressure [3]. In a large U.S. specialty-level analysis, 19.1% of neurosurgeons faced a malpractice claim each year, compared with 7.4% of all physicians overall and 2.6% in psychiatry [1]. In a later National Practitioner Data Bank analysis, neurosurgery also had the highest rate of paid malpractice claims, at 53.1 claims per 1000 physician-years, together with the highest mean payment, at US$469,222 [2]. Litigation analyses in neurosurgery have identified procedural error, delayed diagnosis, and failure to treat as recurrent allegations [4,5], and insurer data indicate that communication problems contribute to neurosurgical claims [6].

Patient complaints and medical disputes are increasingly recognized not only as administrative burdens but also as patient- and family-reported signals of quality deficits, patient-safety risk, and relational breakdown in health care. Systematic analyses of health care complaints have shown that complaints commonly cluster around clinical care, management processes, and interpersonal behavior, and that they can reveal “hot spots” and “blind spots” that may be difficult to detect through staff reporting or routine incident-monitoring systems [7,8]. Complaint records have also been linked to physicians’ risk-management experiences and malpractice risk, indicating that complaints may function as early warning signals rather than merely retrospective expressions of dissatisfaction [9].

In China, medical disputes are a particular concern in tertiary public hospitals, where they have been attributed to interacting system incentives, heavy workload, damaged doctor–patient trust, and limited formal dispute-resolution pathways [10,11]. In a nationwide survey of 22,213 physicians from 144 tertiary public hospitals, 33.48% reported medical disputes and 48.52% verbal violence in the previous 12 months, and this exposure was associated with burnout [12]. Dispute handling follows a national framework. The 2018 Regulation on the Prevention and Handling of Medical Disputes allows disputes to be resolved by negotiation between the hospital and the patient, people’s mediation, administrative mediation, or litigation [13]. The 2019 Measures for the Administration of Complaints in Medical Institutions require hospitals at the secondary level and above to operate a complaint-management office [14]. Under a first-complaint responsibility rule, the department that receives a complaint must record it and either resolve it on the spot or refer the patient to that office. Complaints that need investigation must receive a response within 5 working days, or within 10 working days when several departments are involved. Complaints that cannot be settled in the hospital are directed to mediation or litigation [14]. Studies from Guangdong and Gansu provinces reported that third-party mediation had become the main route for settling medical disputes [15,16]. A recent comparison of complaint systems in China, the United States, the United Kingdom, Germany, and Japan found that system design strongly influenced the efficiency and fairness of dispute resolution [17]. In a Chinese tertiary hospital, a first complaint therefore enters a documented institutional pathway, and escalation corresponds to movement from department-level handling to hospital-level intervention, formal mediation, or legal and regulatory processes.

Despite the growing literature on complaints, malpractice claims, and medical disputes, existing prediction work has mainly focused on practitioner-level or regulator-level risk rather than the complaint event itself [18,19]. Australian national studies showed that complaint recurrence was highly concentrated, with 3% of doctors accounting for 49% of complaints, and the Predicted Risk Of New Event (PRONE) score was subsequently developed to estimate doctors’ future complaint risk using routinely collected administrative variables such as specialty, sex, number of previous complaints, and time since the last complaint [18,20]. A recent scoping review also concluded that risk identification and prediction of complaints and misconduct against health practitioners are multifactorial, context-specific, and still in need of more practical tools [19]. However, these approaches do not directly address the event-level management question faced by hospitals after an initial complaint has already been recorded: which complaint events are more likely to escalate into high-grade disputes?

For this management question, the timing of risk estimation is critical. At the first formal complaint, the hospital has already detected a conflict signal, but the subsequent trajectory and final severity of the event may still be uncertain. Prediction-model reporting and risk-of-bias guidance emphasize that the target population, outcome, candidate predictors, and timing of predictor measurement should be clearly defined [21,22]. This requirement is particularly important in complaint and dispute datasets because many variables that appear predictive, such as final length of stay, final total cost, mediation outcome, post-complaint deterioration, or final dispute grade, may only become available after the event has evolved. Including such variables in a model intended for first-complaint decision-making would create information leakage and could produce overly optimistic performance estimates without real operational usefulness [23]. A model for complaint escalation should therefore be anchored to an explicit prediction timepoint and restrict predictors to information available by then. The first documented complaint provides such a time zero, at which hospital administrators, clinicians, and risk-management staff can still intervene through senior review, structured communication, documentation, and mediation preparation. The early warning concerns the escalation of a complaint that has already been made, not the occurrence of a complaint.

In this study, we developed and temporally validated an interpretable first-complaint-time early-warning risk-stratification model for high-grade escalation among neurosurgical complaint events at a tertiary hospital. The model and nomogram are not intended to replace professional judgment or label patients and families, but to support early triage and structured complaint management for events with higher escalation risk.

## 2. Methods

### 2.1. Study Design, Setting, and Reporting

We conducted a single-center retrospective model-development and temporal-validation study using routinely collected neurosurgical complaint-management records from the Second Xiangya Hospital. The study period included complaint events recorded from January 2016 to December 2025. The target use of the model was early-warning risk stratification after an initial neurosurgical complaint had already been documented. Therefore, the study was designed to estimate the likelihood of subsequent high-grade dispute escalation among complaint events, rather than to predict the occurrence of any complaint among all neurosurgical admissions.

The study was reported in accordance with the TRIPOD + AI statement for prediction-model studies that use regression or machine-learning methods and the RECORD statement for studies using routinely collected health data [24,25]. The completed TRIPOD + AI checklist is provided in File S1. Methodological decisions regarding participant selection, predictor definition, outcome definition, and analysis were also informed by the PROBAST framework for prediction-model risk-of-bias assessment. Particular attention was given to the temporal relationship between candidate predictors and the outcome, because use of post-complaint or post-outcome information can introduce information leakage in prediction studies. The outcome should be interpreted as institutional dispute-management severity rather than legal fault or clinical negligence. Because each final grade was assigned by a single adjudicator, outcome misclassification and institutional subjectivity remain possible; the reproducibility of the grades was therefore assessed by independent re-grading of a sample of events (Section 2.5).

### 2.2. Data Sources and Unit of Analysis

The analysis used de-identified event-level data extracted from institutional complaint-management records and linked clinical-administrative variables. The unit of analysis was the complaint event. The research dataset contained an event-level identifier but no linkable patient or treating-team identifier. Each event record contained administrative information, clinical context, communication-related documentation, sociodemographic information, complaint dates, and final event severity grading. The administrative abstraction date was retained only for source tracing and data management. It was not used as the prediction timepoint. For the primary model, the model-ready dataset was reconstructed using only variables documented on or before the first complaint date.

### 2.3. Participants and Eligibility Criteria

Eligible records were neurosurgical complaint events documented during the study period. Events were included if they met all of the following criteria: documented involvement of neurosurgical care, a recorded first complaint date, an available final event severity grade, and sufficient dated information to determine the primary candidate predictors at the first-complaint timepoint.

**Figure 1:**
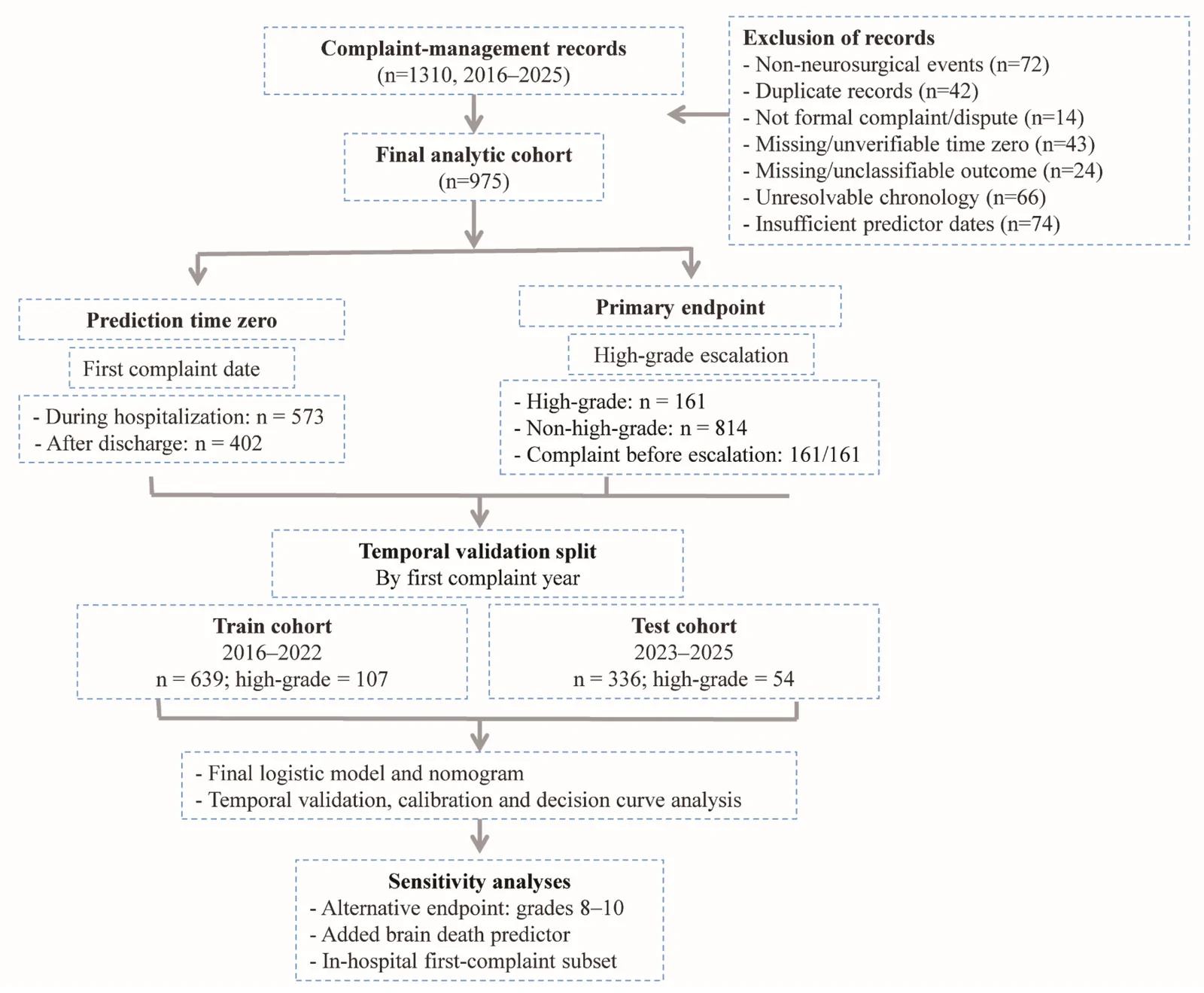
Study cohort and temporal validation design. The initial source screen identified 1310 complaint-management records from the Second Xiangya Hospital (2016–2025). The final analytic cohort included 975 eligible neurosurgical complaint events. After eligibility screening and source-record verification, no further events were excluded for missing first complaint date, final event severity grade, or primary model predictors, and no imputation was performed. The primary model used predictors available at or before time zero. The endpoint was high-grade complaint escalation (*n* = 161; non-high-grade, *n* = 814), with all high-grade events preceded by a complaint record. The cohort was split by first complaint year into training (2016–2022; *n* = 639; high-grade, *n* = 107) and test cohorts (2023–2025; *n* = 336; high-grade, *n* = 54).

The initial source screen identified 1310 complaint-management records from 2016 to 2025. Exclusion was applied hierarchically to create mutually exclusive categories. We excluded 335 records: non-neurosurgical complaint events (*n* = 72), duplicate event records or multiple records referring to the same episode (*n* = 42), records without a formal complaint signal (*n* = 14), missing or unverifiable first complaint date (*n* = 43), missing final event severity grade or inability to classify high-grade versus non-high-grade status (*n* = 24), unresolvable chronological inconsistency after source-record checking (*n* = 66), and insufficient dated information to reconstruct required time-zero candidate predictors (*n* = 74). Twelve records met both the missing first complaint date criterion and the chronological inconsistency criterion; these were assigned to the missing first complaint date category because time zero could not be defined. After exclusions, the final analytic cohort contained 975 eligible neurosurgical complaint events recorded in the Department of Neurosurgery, of which 161 (16.5%) reached high-grade escalation (Figure 1). Post-discharge first complaints were retained in the main cohort because the operational prediction question remained the same once a first complaint had been documented.

### 2.4. Time Zero and Target Early-Warning Question

The prediction timepoint, or time zero, was defined as the date of the first documented formal complaint or dispute signal. The target early-warning question was: among neurosurgical complaint events with an initial complaint already recorded, what is the probability of subsequent high-grade dispute escalation? The model was intended for complaint-management triage at the first complaint, when hospital administrators and clinicians may still intervene through senior review, structured communication, documentation, mediation preparation, and administrative coordination. The model therefore warns of the escalation of a complaint that has already been made; it does not predict whether a complaint will occur.

### 2.5. Outcome Definition

The primary outcome was episode-level high-grade dispute escalation, defined as a final event severity grade of 7–10 versus 1–6 on the hospital’s 10-grade complaint severity scale. The scale is part of routine complaint management and was not developed for this study. Each grade reflects the level at which an event had to be handled. Grades 1–3 denote complaints resolved at first contact, by a written reply from the complaint office, or by a departmental written clarification. Grades 4–6 denote complaints that required department-level review, senior departmental involvement, or coordination by the complaint office, without a hospital-level decision, formal mediation, compensation decision, or legal involvement. Grade 7 denotes hospital-level intervention, such as a hospital-led multidisciplinary review or a hospital-level written decision. Grade 8 denotes the initiation of formal mediation or a settlement or compensation process; grade 9 legal, regulatory, or police involvement; and grade 10 a final high-consequence legal, administrative, or institutional outcome. High-grade escalation therefore denotes handling at hospital level or beyond. The full criteria and the source documents required for each grade are given in Table S1. A sensitivity outcome was defined as grades 8–10 versus grades 1–7 to evaluate model performance under a stricter definition of high-grade escalation. The distribution of severity grades, overall and by year, is summarized in Table S2.

Grades were assigned during routine complaint management rather than retrospectively for this study. Throughout 2016–2025, the final grade was assigned at the final assessment of each event by the director of the hospital complaint-management office, with input from the responsible clinical department when needed, and the grading criteria were not revised during this period. The director had access to the complete complaint file, including the communication records from which the communication-related predictors were later abstracted, so grading was not blinded to these predictors. Each event was graded once by a single adjudicator, and the model-development team used the recorded final grade and did not reassign grades. To assess the reproducibility of the grades, a random sample of 150 events, stratified by recorded outcome status (45 high-grade and 105 other events), was re-graded retrospectively in 2026 by two staff members who were familiar with the hospital’s complaint-grading criteria and had not been involved in the original grading. Each re-grader applied the criteria in Table S1 independently to the complete complaint file and was unaware of the recorded grade, the other re-grader’s grades, and the study-coded predictor values. Agreement among the recorded grade and the two re-grades was estimated with Fleiss’ κ for the 10-level grade and for the binary outcome, with 95% confidence intervals from 2000 bootstrap resamples of the 150 events; the re-grades were used only for this assessment. For high-grade events, the escalation date was recorded and used to describe the interval from the first complaint to escalation. All included events had completed the final institutional event-level assessment by the database lock date.

### 2.6. Candidate Predictors and Time-Availability Rules

Candidate predictors were restricted to information documented on or before the first complaint date. A variable was coded as present for the primary model only when the corresponding event or feature was documented on or before time zero. Features documented only after the first complaint date were not treated as available predictors in the primary model. Clinical candidate predictors included ICU admission before the first complaint, reoperation before the first complaint, nursing-related friction before the first complaint, invasive manipulation failure before the first complaint, and brain death before the first complaint. Invasive manipulation failure was defined as an unsuccessful attempt at external ventricular drain placement, lumbar drain placement, central venous catheter placement, or lumbar puncture. It was coded only when the clinical record stated that the intended access or catheter placement was not achieved and the procedure was abandoned or required a new attempt. Complications after a successfully completed procedure were not coded as manipulation failure, and surgical procedures such as craniotomy were not included; reoperation was recorded separately. Brain death before the first complaint was not included in the primary model because it represents an extreme terminal clinical state that could dominate prediction and limit general applicability; it was evaluated only in sensitivity analysis. The cumulative hospitalization cost of at least 45,000 CNY before the first complaint was also considered. Cost was counted from admission to the first complaint date or, for complaints first made after discharge, over the whole admission, and the cut-off lay close to the cohort median (53.7% of events at or above it). The nonterminal clinical harm count, defined as the number of the four features (ICU admission, reoperation, nursing-related friction, and invasive manipulation failure) present before the first complaint, was used descriptively.

Communication-related predictors were derived from dated complaint and communication records. The record of the first complaint was written by the staff member who received it. A communication-context factor before the first complaint was defined as the presence of at least one non-anger communication or emotional-conflict feature documented on or before the first complaint date, including multiple relatives involved, relatives’ disagreement, lack of communication, open audio or video recording, offensive language, depressed mood, reasonable doubt, or defensive attitude. Anger documented at the first complaint communication was coded as present when the first-complaint record described the complainant with expressions such as emotional agitation, strong dissatisfaction, or an on-site argument. Anger was modeled separately because it represented a distinct early conflict signal. Both variables were abstracted by a single study abstractor who was not blinded to the final grade. For the 150 events in the re-graded sample (Section 2.5), a second abstractor who was unaware of the final grade and of the original codes re-coded anger and each component of the communication-context factor from the same records, and agreement with the original codes was estimated with Cohen’s κ. Only the composite communication-context indicator was retained in the analytic dataset; the individual components were not retained, so item-level analyses were not possible. Definitions of the components are given in Table S3.

Sociodemographic variables included education level, occupation, age group, residence, and sex. A sociodemographic support-needs profile was defined as the presence of at least one of the following features: primary school education or below, freelance or unemployed occupation, age 61 years or older, or residence outside the province. The profile was not offered as a candidate predictor, because using it to flag complaint events could stigmatize patients and families with greater support needs; it was used in descriptive and fairness analyses. Variables not reliably available at time zero were excluded from the primary predictor set. These included final total cost, final length of stay, post-complaint brain death or reoperation, post-complaint clinical harm, post-complaint mediation, and final event severity grade. Candidate variable definitions and modeling roles are listed in Table S4. The model-ready time-zero availability summary is provided in Table S5.

### 2.7. Missing Data and Data Cleaning

After eligibility screening and source-record verification, the final analytic cohort contained complete information for the primary outcome, first complaint date, and all primary model predictors; therefore, no additional events were excluded from the primary analysis for missing key model variables. No imputation was performed. For date-based predictors, events were coded as positive only when the feature date was present and occurred on or before the first complaint date; otherwise, the feature was coded as absent for the primary time-zero model. The records did not indicate whether a feature had been assessed, so an undocumented feature could not be distinguished from a truly absent one; the effect of this coding was examined in a misclassification analysis (Section 2.14). Missing escalation dates occurred only for non-high-grade events and were not treated as missing outcome data.

### 2.8. Descriptive Analysis

Baseline characteristics at first complaint were summarized overall and by primary outcome status. Continuous variables were reported as medians and interquartile ranges because several timing and count variables were not normally distributed. Categorical variables were reported as counts and percentages. Standardized mean differences (SMDs) were used to quantify differences between non-high-grade and high-grade groups. For categorical variables with more than two levels, an omnibus SMD was calculated as the Mahalanobis distance between the category-proportion vectors of the two groups.

### 2.9. Primary Early-Warning Model Development

Logistic regression was selected as the primary modeling approach. The final primary model included four non-sociodemographic predictors available at the first-complaint timepoint: ICU admission before the first complaint, invasive manipulation failure before the first complaint, communication-context factor before the first complaint, and anger documented at the first complaint communication.

Seven binary candidate predictors, each assessed on or before time zero, were considered: ICU admission, reoperation, nursing-related friction, invasive manipulation failure, communication-context factor, anger at the first complaint, and cumulative cost of at least 45,000 CNY. Predictor selection was data-driven. Candidates with *p* < 0.05 in univariable logistic regression were entered into a multivariable model, and candidates with *p* ≥ 0.05 in the multivariable model were removed. Because univariable screening was originally performed in the full cohort (Table S6), the whole procedure was repeated in the 2016–2022 training cohort alone, and all 127 combinations of the seven candidates were compared by Akaike information criterion (AIC) in the training cohort (Table S7). For each event, the model estimated the probability of high-grade dispute escalation using the logistic function:

$$p=\frac{1}{1+\exp\left(-\mathrm{LP}\right)}$$

where *LP* is the linear predictor from the fitted logistic regression model. Regression coefficients, standard errors, adjusted odds ratios, 95% confidence intervals, *p* values, and a simplified point score were reported. The point score was derived by dividing each coefficient by the smallest coefficient, that of ICU admission, and rounding to the nearest integer. All performance analyses used the original regression-based estimated probabilities rather than the simplified point score. For clinical presentation, the final logistic model was additionally displayed as a nomogram, in which the points for each predictor are proportional to its regression coefficient; its performance was evaluated using the same model-level discrimination, calibration, prediction-error, temporal-validation, and decision-curve analyses described below.

### 2.10. Apparent Performance and Internal Validation

Model performance was assessed using discrimination, calibration, and overall prediction error. Discrimination was evaluated using the area under the receiver operating characteristic curve (AUC). Because the primary outcome was imbalanced, with high-grade events representing a minority of the cohort, average precision was also reported as the area under the precision-recall curve (PR-AUC), which is recommended as an informative complement to ROC analysis in imbalanced binary classification settings [26]. Overall prediction error was assessed using the Brier score [27].

Calibration was evaluated using grouped calibration plots comparing mean estimated risk with observed event rates across equal-count risk groups. The calibration intercept and calibration slope were estimated using logistic recalibration methods. A reference threshold was derived using the Youden index, and sensitivity, specificity, positive predictive value, and negative predictive value were reported descriptively at this threshold. This threshold was used to describe operating characteristics rather than to define a mandatory clinical decision rule. Internal validation of apparent discrimination was performed using bootstrap resampling. A bootstrap 95% confidence interval for the apparent AUC was calculated using 1000 resamples. Optimism correction for AUC was performed using 1000 bootstrap resamples, with the complete predictor-selection procedure repeated in each resample, and the optimism-corrected AUC was reported. These analyses were used to quantify internal overfitting, whereas temporal validation was treated as the primary out-of-time validation analysis.

### 2.11. Temporal Validation

Temporal validation was performed according to the year of the first complaint. Events from 2016 to 2022 were assigned to the training cohort, and events from 2023 to 2025 were assigned to the temporal test cohort. The primary logistic regression model was fitted in the training cohort and then applied unchanged to the temporal test cohort. Temporal validation performance was evaluated using AUC, PR-AUC, Brier score, sensitivity, specificity, positive predictive value (PPV), and negative predictive value (NPV). The classification threshold used in the temporal test cohort was derived from the Youden index in the training cohort and then applied without modification to the test cohort. Operating characteristics were also reported at fixed thresholds of 0.10, 0.15, and 0.20. Uncertainty in the temporal AUC, PR-AUC, Brier score, and calibration intercept and slope was estimated from 2000 bootstrap resamples of the test cohort, and Wilson 95% confidence intervals were calculated for sensitivity, specificity, PPV, and NPV.

Temporal calibration was assessed in the test cohort using calibration intercept, calibration slope, and grouped calibration plots. For grouped calibration, the apparent full-cohort analysis used deciles of estimated risk, whereas the temporal test cohort used five equal-count groups because of the smaller test-sample size.

### 2.12. Risk Stratification

For clinical and administrative interpretability, estimated probabilities from the primary model were categorized into three exploratory risk strata: low risk (<0.10), intermediate risk (0.10 to <0.20), and high risk (≥0.20). Observed high-grade event rates were calculated within each stratum. These thresholds were selected to create practical complaint-management categories rather than to optimize a single statistical criterion. The risk strata were intended to support prioritization of senior review, structured family communication, documentation, and administrative coordination. These thresholds have not been validated as decision rules and should be regarded as exploratory until prospective external validation is available.

### 2.13. Decision Curve Analysis

Clinical utility was evaluated using decision curve analysis. Net benefit was calculated across threshold probabilities from 0.05 to 0.50 and compared with two default strategies: treating all complaint events as high risk and treating no events as high risk [28]. Net benefit was calculated as follows:

$$\mathrm{Net}\;\mathrm{benefit}\;=\frac{\mathrm{TP}}{N}-\frac{\mathrm{FP}}{N}\times \frac{p_{t}}{1-p_{t}}$$

where *TP* is the number of true positives, *FP* is the number of false positives, *N* is the number of events, and *p_t_* is the threshold probability. Decision curves were produced for both the apparent cohort analysis and the temporal test cohort. In our hospital, a complaint judged to be high risk is followed by a meeting between a senior physician (associate chief physician or above) and the patient or family within 48 h, with notification of the complaint office for early coordination. This response was taken as the action triggered by a positive classification. A threshold probability *p_t_* implies accepting up to (1 − *p_t_*)/*p_t_* such responses without subsequent escalation for each escalation correctly identified, for example, 4 at *p_t_* = 0.20.

### 2.14. Sensitivity Analyses

Several supplementary analyses were performed. First, the primary endpoint model was refit after adding brain death before the first complaint as an additional predictor to examine the influence of an extreme terminal clinical marker. Second, the model was evaluated using the stricter high-grade endpoint of grades 8–10. For this alternative endpoint, the primary predictor set was refit, and both apparent and temporal test performance were reported.

Exploratory machine-learning comparisons were conducted within the same temporal-validation framework. LASSO logistic regression, elastic-net logistic regression, random forest, and gradient boosting models were trained in the 2016–2022 cohort and evaluated in the 2023–2025 test cohort. Regularized regression models used cross-validation within the training cohort. Tree-based models used grid search within the training cohort. Model comparisons emphasized temporal test AUC, PR-AUC, and Brier score rather than apparent performance, and detailed test-cohort results are provided in Table S8. Permutation importance was estimated in the temporal test cohort by measuring the decrease in AUC after permuting each predictor (Table S9).

Four further analyses were performed. First, to examine the effect of coding undocumented features as absent and of possible selective recording, anger and the communication-context factor were each perturbed in 200 simulated datasets. Three scenarios were applied, each at proportions of 10% and 20%: random reclassification of events coded as absent to present, irrespective of outcome; the same reclassification restricted to events that did not escalate; and reclassification of high-grade events coded as present to absent. The model was refitted in each simulated dataset, and the adjusted odds ratio and temporal AUC were recorded. Second, models omitting the communication-context factor, anger, or both were fitted in the training cohort and evaluated in the test cohort (Table S10). Third, observed and mean predicted event rates, observed-to-expected (O/E) ratios, and AUCs were compared across age groups, places of residence, and support-needs profiles. Fourth, the model was refitted with the support-needs profile added to quantify the performance forgone by excluding it (Table S11).

### 2.15. Statistical Analyses

All data cleaning, statistical analyses, model development, validation, and figure generation were performed using Python 3.13.7 (Python Software Foundation, Wilmington, DE, USA). Logistic regression analyses were conducted using statsmodels 0.14.6, and discrimination, calibration-related summaries, Brier scores, regularized regression, random forest, gradient boosting, cross-validation, grid search, and permutation importance were performed using scikit-learn 1.8.0. The analytic workflow followed standard clinical prediction-model and biomedical machine-learning guidance.

### 2.16. Ethics Approval

The study was approved by the Clinical Research Ethics Committee of the Second Xiangya Hospital, Central South University, on 27 February 2026 (Approval No. 2022251). The approval covers the retrospective analysis of complaint events recorded from January 2016 to December 2025. At admission, all patients or their legal representatives signed the hospital’s general written authorization for the use of de-identified medical records for research.

## 3. Results

### 3.1. Study Population and Endpoint Distribution

Of 1310 complaint-management records screened at the Second Xiangya Hospital from 2016 to 2025, 335 were excluded during hierarchical eligibility screening and source-record verification, leaving 975 eligible neurosurgical complaint events in the final analytic cohort (Figure 1). The primary endpoint was high-grade dispute escalation, defined as event severity grades 7–10. Overall, 161 events (16.5%) were classified as high-grade and 814 (83.5%) as non-high-grade. The annual proportion of high-grade events ranged from 12.6% to 23.3% without a temporal trend (odds ratio per year, 1.01; *p* = 0.69; Table S2). In the re-graded sample of 150 events, the recorded grade and the two re-grades were identical for 136 events (90.7%), and no two grades differed by more than one level. Fleiss’ κ was 0.928 (95% CI, 0.889–0.959) for the 10-level grade and 0.958 (95% CI, 0.911–0.990) for high-grade escalation, on which all three gradings agreed for 146 events (97.3%). The second abstractor’s codes agreed with the original codes for 144 events (96.0%) for anger (Cohen’s κ, 0.896; 95% CI, 0.810–0.967) and for 142 events (94.7%) for the communication-context factor (κ, 0.888; 95% CI, 0.806–0.958). The first complaint date was used as time zero for all primary analyses (Figure S1). The first complaints occurred during hospitalization in 573 events (58.8%) and after discharge in 402 events (41.2%). All high-grade events had a recorded first complaint before escalation. For temporal validation, events from 2016–2022 formed the training cohort (*n* = 639, high-grade events = 107), and events from 2023–2025 formed the test cohort (*n* = 336, high-grade events = 54). High-grade escalation occurred a median of 25 days (interquartile range [IQR], 14–33) after the first complaint and within 7 days in 18 of the 161 high-grade events (11.2%); final grades were assigned a median of 67 days (IQR, 41–92) after the first complaint.

### 3.2. Baseline Characteristics at First Complaint

Baseline characteristics at the first complaint are summarized in Table 1. The interval from admission to first complaint was similar between high-grade and non-high-grade events. High-grade events had a greater burden of complaint-time risk markers, including a higher nonterminal clinical-harm count and higher frequencies of ICU admission, invasive manipulation failure, communication-context factors, and anger documented at the first complaint communication, with SMDs ranging from 0.457 to 0.736. Age group, residence, and sociodemographic support-needs profile also showed moderate standardized differences between groups. Univariable logistic analyses for candidate predictors are provided in Table S6. Brain death before the first complaint was uncommon (*n* = 22) and was not more frequent among high-grade events (1.2% vs. 2.5%); because of the small numbers, this difference was not interpreted.

### 3.3. Final Primary Model

**Figure 2:**
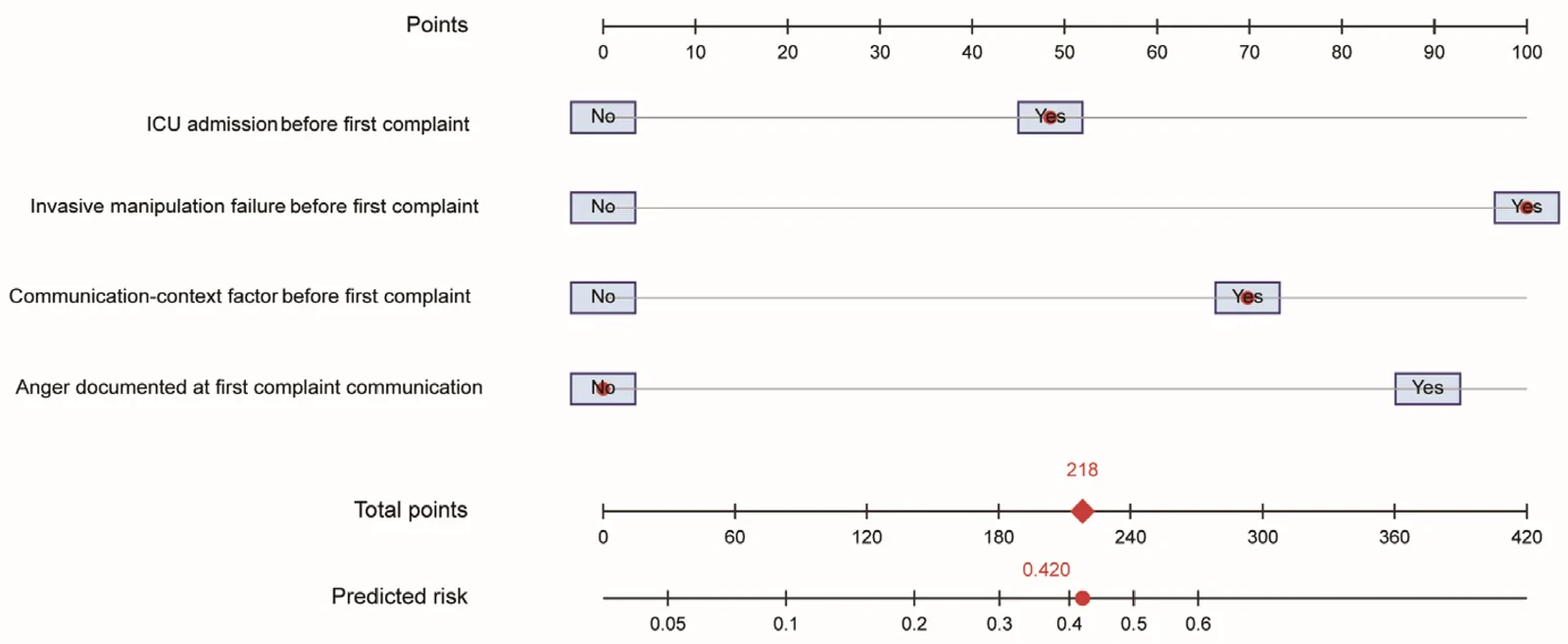
Nomogram for the primary first-complaint-time model. For each predictor, the presence or absence of the feature is mapped to the points scale, with points proportional to the regression coefficient (invasive manipulation failure = 100 points); total points are then mapped to the estimated probability of high-grade dispute escalation. The red markers show an illustrative example only and do not affect model performance estimation. With ICU admission, invasive manipulation failure, and the communication-context factor present and anger absent, the total is 48.5 + 100 + 69.8 = 218 points, corresponding to *LP* = −0.322 and an estimated probability of 0.42 (see the equation below Table 2).

The final primary logistic model included four non-sociodemographic predictors (Table 2, Figure 2 and Figure S2). In the full cohort, six candidates passed univariable screening; reoperation (*p* = 0.20) and nursing-related friction (*p* = 0.32) were not independently associated with high-grade escalation in the multivariable model and were removed. Repeating the procedure in the training cohort alone selected the same four predictors: nursing-related friction did not pass univariable screening (*p* = 0.26), and reoperation was removed in the multivariable step (*p* = 0.18). Among all 127 candidate combinations, the selected model had the lowest training-cohort AIC (479.1); adding reoperation gave a similar AIC (479.4) and the same temporal AUC (0.759), and adding nursing-related friction or cost did not materially improve temporal performance (Table S7). The same predictors were selected with thresholds of 0.10 at both steps. When the full selection procedure was repeated in 1000 bootstrap samples, invasive manipulation failure, anger, and the communication-context factor were selected in 99–100% of samples and ICU admission in 92%, whereas reoperation and nursing-related friction were selected in 27% and 17%, respectively. ICU admission before the first complaint was associated with higher odds of high-grade escalation (adjusted OR, 1.96; 95% CI, 1.34–2.87; *p* < 0.001). Invasive manipulation failure before the first complaint was independently associated with high-grade escalation (adjusted OR, 4.01; 95% CI, 2.66–6.05; *p* < 0.001). The communication-context factor before the first complaint was also associated with high-grade escalation (adjusted OR, 2.64; 95% CI, 1.68–4.14; *p* < 0.001), as was anger documented at the first complaint communication (adjusted OR, 3.46; 95% CI, 2.34–5.11; *p* < 0.001).

### 3.4. Model Performance and Temporal Validation

**Figure 3:**
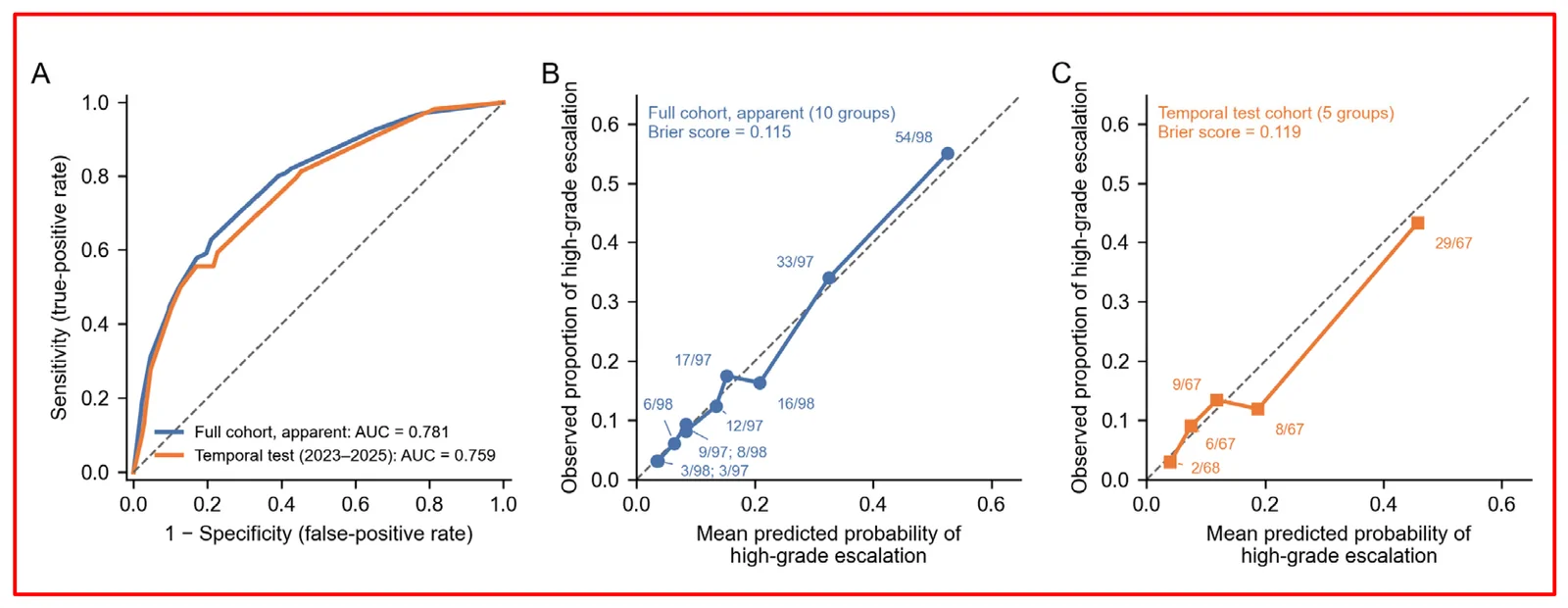
Discrimination and calibration of the primary early-warning model. (**A**) Receiver operating characteristic curves for the primary endpoint showed AUCs of 0.781 in the full apparent cohort and 0.759 in the temporal test cohort. (**B**,**C**) Calibration plots comparing the mean predicted probability with the observed proportion of high-grade escalation in equal-count groups of predicted risk in the full apparent cohort ((**B**); 10 groups) and the temporal test cohort ((**C**); 5 groups). Labels give the number of high-grade events/number of events in each group. Brier scores were 0.115 for the full apparent cohort and 0.119 for the temporal test cohort, with a temporal calibration intercept of −0.132 and slope of 0.839.

Model performance is summarized in Table S12 and displayed in Figure 3A–C. In the full analytic cohort, the apparent AUC was 0.781 (bootstrap 95% CI, 0.741–0.818), and the optimism-corrected AUC, with the complete selection procedure repeated in each bootstrap sample, was 0.774. The apparent PR-AUC was 0.417 and the Brier score was 0.115. At the apparent Youden threshold of 0.192, sensitivity was 0.627, specificity was 0.790, positive predictive value was 0.371, and negative predictive value was 0.915.

In temporal validation, the model trained on the 2016–2022 cohort achieved an AUC of 0.759 (95% CI, 0.687–0.826) in the 2023–2025 test cohort, with a PR-AUC of 0.364 (95% CI, 0.263–0.496) and a Brier score of 0.119 (95% CI, 0.096–0.144). Applying the training-derived threshold of 0.209 to the temporal test cohort yielded a sensitivity of 0.593 (95% CI, 0.460–0.713), a specificity of 0.773 (95% CI, 0.721–0.818), a PPV of 0.333 (95% CI, 0.247–0.432), and an NPV of 0.908 (95% CI, 0.865–0.939). Because the four binary predictors produce only 15 distinct predicted probabilities in the test cohort, none of them between 0.149 and 0.209, thresholds of 0.15 and 0.20 classified the test cohort identically to the training-derived threshold (Table S12). At a threshold of 0.10, sensitivity was 0.815 and PPV 0.254, with 51.5% of events flagged. These operating characteristics indicate moderate early-warning performance at a descriptive threshold rather than a definitive individual-level classification rule. The temporal calibration intercept was −0.132 (95% CI, −0.500 to 0.203) and the calibration slope was 0.839 (95% CI, 0.579–1.158); grouped calibration summaries are provided in Table S13. The slope below 1 suggests that predictions were somewhat too extreme; in the high-risk stratum, the mean predicted risk was 39.2% and the observed rate 33.3% (Table S14). Recalibration may therefore be needed before prospective implementation.

### 3.5. Risk Stratification and Decision Curve Analysis

Risk stratification based on the primary-model estimated probability separated the cohort into three exploratory risk groups (Table S14 and Figure S3). In the full analytic cohort, the observed high-grade event rate was 5.8% in the low-risk group (<0.10; 29/497), 16.6% in the intermediate-risk group (0.10 to <0.20; 37/223), and 37.3% in the high-risk group (≥0.20; 95/255). In the temporal test cohort, the corresponding observed rates were 6.1% (10/163), 15.6% (12/77), and 33.3% (32/96). Decision curve analysis showed positive net benefit across clinically plausible lower-to-moderate threshold probabilities in both the full apparent and temporal test cohorts (Figure S4A,B and Table S15). At a threshold probability of 0.20, the net benefit in the temporal test cohort was 0.048, compared with −0.049 for treating all events as high risk. For the response described in Section 2.13, this corresponds to two unnecessary meetings for each escalation correctly flagged, within the four accepted at this threshold.

### 3.6. Sensitivity Analyses

Adding brain death before the first complaint, a terminal clinical marker, produced a similar temporal test AUC of 0.761, so this variable was kept out of the primary model to avoid over-reliance on a severe late-stage clinical state (Table S16). Sensitivity-analysis ROC curves showed an AUC of 0.781 in the full apparent cohort and 0.759 in the temporal test cohort for the primary endpoint definition of grades 7–10 (Figure S5A). Under the stricter alternative endpoint definition of grades 8–10, there were 85 events overall, 59 in the development cohort, and 26 in the temporal test cohort; the corresponding AUCs were 0.757 and 0.722, respectively (Figure S5B). Performance was weaker when the first complaint was made during hospitalization, which applied to 573 of 975 events (58.8%). In this subgroup, the temporal AUC was 0.687 and the PR-AUC 0.262 when the model was refitted within the subgroup, and 0.691 and 0.273 when the primary model was applied unchanged. For complaints first made after discharge, the primary model achieved an AUC of 0.842 and a PR-AUC of 0.534 (Table S16).

In the misclassification analysis, the adjusted odds ratio for anger ranged from 1.46 to 2.88 and that for the communication-context factor from 1.12 to 2.51, compared with 3.46 and 2.64 in the primary model. The largest reductions occurred when anger was assumed to be under-recorded in events that did not escalate or the communication-context factor was over-recorded in escalated events. Across all scenarios, the temporal AUC ranged from 0.720 to 0.754. Without the communication-context factor, anger, or both, the temporal AUC was 0.730, 0.712, and 0.682, respectively (Table S10).

In the temporal test cohort, O/E ratios across age groups, places of residence, and support-needs profiles ranged from 0.72 to 1.05 (Table S11). The exceptions were children aged 0–10 years (O/E, 0.16; 1 event among 37) and events without the support-needs profile (O/E, 0.38; 4 events among 71), in which the model overestimated risk. Adding the support-needs profile to the model increased the temporal AUC from 0.759 to 0.778 and reduced this overestimation (O/E, 0.68).

## 4. Discussion

In this study, we developed an interpretable early-warning model for escalation of neurosurgical complaint events. The study design was retrospective and single-center, and model performance was assessed using temporal validation. From a hospital-management perspective, the key methodological feature was the explicit use of the first documented complaint as the operational index timepoint. At that point, the organization has already identified a conflict signal, but the downstream trajectory of the event remains modifiable through managerial and clinical intervention. Using this framework, we found that 161 of 975 neurosurgical complaint events (16.5%) progressed to high-grade escalation. The final nomogram incorporated four non-sociodemographic predictors available at or before the first complaint: ICU admission, invasive manipulation failure, a pre-complaint communication-context factor, and anger documented during the first complaint encounter. Accordingly, the main contribution of this study is not only a prediction model, but also the definition of a practical complaint-escalation risk stratification task that is aligned with real-world hospital complaint-management workflows.

The model demonstrated moderate discriminatory performance and clinically meaningful risk enrichment. In the apparent cohort, the AUC was 0.781 and the optimism-corrected AUC was 0.774. In the 2023–2025 temporal validation cohort, the AUC remained 0.759 (95% CI, 0.687–0.826), with a PR-AUC of 0.364 and a Brier score of 0.119. These findings suggest that routinely available first-complaint-time information can support operational triage, even though the model is not sufficiently accurate to function as a stand-alone classification rule. The observed escalation gradient across the exploratory strata was also managerially relevant: high-grade escalation occurred in 5.8% of low-risk events, 16.6% of intermediate-risk events, and 37.3% of high-risk events. This separation may help prioritize senior review and early communication for a subgroup of complaint events that consume disproportionate governance resources. With a PPV of about one-third at the 0.20 threshold, however, two of every three flagged events did not escalate, so a flag should prompt review rather than a presumption of escalation.

These findings should be interpreted within the broader context of complaint surveillance and specialty-level medicolegal risk. Prior work has shown that patient complaints are not merely expressions of dissatisfaction; they are also patient- and family-reported indicators of failures in care processes, communication, and safety surveillance [29,30,31]. In parallel, practitioner-level studies have shown that complaint recurrence is concentrated in a relatively small proportion of clinicians, and tools such as the PRONE score were designed to identify doctors at risk of future formal complaints [18,20]. However, hospitals manage complaint events, not just clinicians. Once the first formal complaint has entered the complaint-management system, the more operationally relevant question is whether that event is likely to escalate and require intensified dispute-management resources. Our study extends the literature by shifting the analytic focus from practitioner recurrence to event-level escalation after complaint onset. This distinction is important for hospital management because the intervention window, response team, and resource allocation logic differ fundamentally between physician monitoring and complaint-event triage.

This event-level approach is especially relevant in neurosurgery, where medicolegal exposure is consistently high. Neurosurgery has one of the highest annual claim rates and the highest paid-claim rate among specialties, and litigation studies show repeated concentration of disputes around procedural error, diagnostic delay, treatment failure, informed-consent problems, and death [4,5,6]. These data are consistent with the clinical predictors retained in our model.

The predictor pattern in the final model is also plausible from a hospital complaint-management perspective. ICU admission before the first complaint may function as a proxy for disease severity, higher uncertainty, repeated family updates, and greater emotional volatility during the care episode. A failed attempt at an invasive procedure, such as drain or catheter placement, is a concrete event that patients or families can attribute to the care team. Anger documented at the first complaint encounter may indicate that the event has already shifted from routine grievance expression toward an adversarial complaint trajectory. The communication-context factor captures antecedent relational strain, including disagreement among family members, perceived inadequate communication, or defensive interaction patterns. Importantly, sociodemographic variables should not be interpreted as markers of “problematic patients.” In hospital-management terms, they are better understood as possible indicators of support needs, including health-literacy support, communication tailoring, or administrative coordination requirements.

These two communication variables, however, require cautious interpretation. They were abstracted from the same complaint and communication records that the complaint-office director could consult when assigning the final grade, and the abstractor was not blinded to that grade. Staff who regarded a complaint as difficult may also have been more likely to record anger. Their associations with escalation may therefore partly reflect how difficult complaints are recorded and graded. When a second abstractor who was unaware of the final grade re-coded 150 events, agreement with the original codes was high (κ, 0.896 for anger and 0.888 for the communication-context factor), so the coding rules could be applied consistently; this check cannot show, however, whether the records themselves were written differently for complaints that staff regarded as difficult. We therefore interpret their odds ratios as associations within a single administrative record system rather than as independent clinical signals. In the misclassification analysis, selective recording reduced these odds ratios substantially, to 1.46 for anger and 1.12 for the communication-context factor in the most extreme scenarios, whereas the temporal AUC remained between 0.720 and 0.754.

Performance was weaker for complaints first made during hospitalization, which accounted for 58.8% of events (temporal AUC, 0.687–0.691), than for complaints first made after discharge (0.842). This matters because early intervention is most feasible while the patient is still in the hospital. A possible explanation is that, during an ongoing admission, the clinical course is still evolving, so information available at the first complaint captures less of what later drives escalation. For in-hospital complaints, the model estimate should therefore be combined with clinical reassessment as the admission unfolds, and future work could test whether updating predictors during the admission improves performance.

A major methodological strength of this study is the strict definition of the prediction timepoint. In complaint and dispute datasets, many highly predictive variables are only observable after the event has progressed, including final length of stay, total hospitalization cost, mediation outcomes, late clinical deterioration, post-complaint brain death, and final dispute grade. If such variables were entered into a model intended for first-complaint decision support, the result would be information leakage and an inflated estimate of apparent model performance [32,33,34,35]. By reconstructing candidate predictors as time-zero-available variables and excluding post-complaint information from the primary model, we prioritized operational validity over maximal statistical fit. This design choice likely reduced model optimism and strengthened the model’s relevance for real-time use in complaint triage. The temporal validation result is therefore especially important. The decline from apparent to out-of-time performance was limited.

The temporal calibration estimates were imprecise (slope, 0.839 [95% CI, 0.579–1.158]; intercept, −0.132 [95% CI, −0.500 to 0.203]), but the slope below 1 and the lower observed than predicted rate in the high-risk stratum (33.3% vs. 39.2%) suggest that the model overestimates risk for events with several risk signals. Before prospective use, we would first update the intercept using recent local data, then re-estimate the calibration slope by logistic recalibration of the linear predictor once enough high-grade events have accrued, and refit individual coefficients only if calibration remains poor [36,37]. Calibration should then be monitored at regular intervals, overall and across patient groups, because complaint patterns, documentation practices, and grading may change over time.

From an implementation standpoint, model output should support, not replace, the judgment of complaint-office staff and clinicians. In our hospital, a complaint judged to be high risk is followed by a meeting between a senior physician (associate chief physician or above) and the patient or family within 48 h, and the complaint office is notified for early coordination. A model-derived high-risk flag would trigger the same two actions. At the 0.20 threshold, 96 of 336 events (28.6%) in the temporal test cohort were flagged, about 32 per year or two to three meetings per month, and about two of every three flags (64 events, about 21 per year) did not precede high-grade escalation. The 0.20 threshold implies accepting up to four such meetings for each escalation correctly identified; the observed ratio was two to one, which is why the model retained a positive net benefit at this threshold. Because a false-positive flag adds a meeting rather than restricting care, its main costs are senior-staff time and administrative workload. These costs are not trivial. Frequent flags could divert complaint-office capacity from events that were not flagged, and staff aware of a flag could respond defensively, for example by ordering additional tests or documenting mainly for self-protection, practices already reported by neurosurgeons facing liability pressure [38]. Flag volume, meeting workload, and the proportion of flagged events that escalate should therefore be monitored, and the threshold adjusted locally. Low- and intermediate-risk events should remain within standard complaint handling and be reassessed if new risk signals emerge.

Several limitations should be acknowledged. First, this was a single-center retrospective study from a tertiary hospital, and model performance may differ across hospitals, regions, specialties, legal environments, and complaint-management systems. External validation and local recalibration are required before the model is adopted in any other institution. Second, the data were derived from complaint-management records and linked clinical-administrative documentation. Such records may be affected by incomplete documentation, variable coding practices, institutional policy changes, and retrospective abstraction bias, and undocumented features were coded as absent. Third, the outcome was based on final event severity grading by a single adjudicator who was not blinded to the communication records. Agreement with two independent re-graders was high in a sample of 150 events, but the re-grading was retrospective and was based on the same complaint files. Although the grading criteria and the adjudicator were unchanged throughout the study period and the annual proportion of high-grade events showed no trend, the grades may still include subjective or administrative judgment. Future studies should evaluate inter-rater reliability of the severity grades prospectively and in other institutions. Fourth, the communication variables were coded by an abstractor who was not blinded to the final grade; agreement with a second abstractor who was unaware of the final grade was high in 150 re-coded events, but the components were not retained in the analytic dataset, so the contribution of individual components could not be assessed. Fifth, the dataset contained no linkable patient or treating-team identifier, so we could not determine whether several complaint events arose from the same patient, family, or team; if such clustering exists, the reported confidence intervals may be too narrow. Sixth, predictors were selected by *p*-value-based screening; although the training-only analysis and the bootstrap repetition of the selection procedure gave consistent results, some optimism may remain. Seventh, the model estimates escalation risk only among recorded complaint events and should not be generalized to all neurosurgical admissions or used to predict complaint occurrence. Eighth, several potentially important predictors were not available or not consistently measurable, including detailed communication quality, patient and family expectations, socioeconomic stressors, legal involvement, media exposure, clinician workload, and real-time institutional response. Ninth, the temporal test cohort contained 336 events and 54 high-grade events, which limits precision for validation estimates and subgroup analyses. Tenth, the model showed moderate discrimination and imperfect temporal calibration. It should therefore be used only as a decision-support tool and not as a definitive classifier. Finally, excluding the support-needs profile from the model did not make its predictions neutral with respect to that profile. The model overestimated risk for events without the profile and for young children. Any implementation should include safeguards to prevent stigmatization, monitor calibration across patient groups, and ensure that higher estimated risk leads to additional support rather than reduced care or defensive management.

Future work should externally validate the model in other hospitals, regions, and clinical specialties, with local recalibration before use. Prospective validation is also needed to determine whether the model performs reliably in real-time complaint-management workflows.

Future studies should also test whether model-guided interventions improve outcomes. Important endpoints may include reduction in high-grade escalation, shorter dispute resolution time, improved patient and family satisfaction, reduced clinician distress, and lower administrative burden. In addition, future datasets should record communication features at the item level, coded independently of the outcome, and capture standardized complaint-handling processes, patient- and family-reported concerns, and institutional response times. These additions may improve risk stratification and make the model more useful for designing targeted interventions.

## 5. Conclusions

In this single-center retrospective study, a model anchored at the first documented complaint provided moderate discrimination and an exploratory risk gradient for high-grade escalation among neurosurgical complaint events. By defining the first complaint date as time zero and excluding post-complaint information, the model is aligned with the point at which hospitals can still act. Its positive predictive value of about one-third means that it should be used to prioritize senior review and early communication, not as a stand-alone classifier or to label patients and families. Before adoption in other hospitals, the model requires external validation and local recalibration, and any implementation should monitor calibration across patient groups.

## Figures and Tables

**Table 1:** Characteristics of neurosurgical complaint events at the first complaint, overall and by escalation status.

Characteristic	Overall (*n* = 975)	Non-High-Grade (*n* = 814)	High-Grade (*n* = 161)	SMD
Days from admission to first complaint	13.0 (10.0–17.0)	13.0 (10.0–17.0)	13.0 (10.0–17.0)	0.053
Nonterminal clinical harm count before complaint	1.0 (0.0–1.0)	1.0 (0.0–1.0)	1.0 (1.0–2.0)	0.736
First complaint during hospitalization	573 (58.8)	481 (59.1)	92 (57.1)	0.039
ICU admission before first complaint	455 (46.7)	350 (43.0)	105 (65.2)	0.457
Reoperation before first complaint	121 (12.4)	88 (10.8)	33 (20.5)	0.269
Nursing-related friction before first complaint	171 (17.5)	133 (16.3)	38 (23.6)	0.182
Invasive manipulation failure before first complaint	157 (16.1)	94 (11.5)	63 (39.1)	0.669
Brain death before first complaint	22 (2.3)	20 (2.5)	2 (1.2)	0.090
Communication-context factor before first complaint	602 (61.7)	470 (57.7)	132 (82.0)	0.548
Anger documented at the first complaint communication	197 (20.2)	124 (15.2)	73 (45.3)	0.694
Cost ≥ 45,000 CNY before first complaint	524 (53.7)	431 (52.9)	93 (57.8)	0.097
Sociodemographic support-needs profile	784 (80.4)	636 (78.1)	148 (91.9)	0.394
Sex				0.066
Male	512 (52.5)	423 (52.0)	89 (55.3)	
Female	463 (47.5)	391 (48.0)	72 (44.7)	
Education level				0.055
College or above	315 (32.3)	260 (31.9)	55 (34.2)	
Middle/high school	352 (36.1)	297 (36.5)	55 (34.2)	
Primary school or below	308 (31.6)	257 (31.6)	51 (31.7)	
Occupation				0.121
Employee institution or retired	418 (42.9)	343 (42.1)	75 (46.6)	
Self-employed	278 (28.5)	239 (29.4)	39 (24.2)	
Unemployed	279 (28.6)	232 (28.5)	47 (29.2)	
Age group				0.288
0–10 years	123 (12.6)	111 (13.6)	12 (7.5)	
11–39 years	339 (34.8)	287 (35.3)	52 (32.3)	
40–60 years	220 (22.6)	187 (23.0)	33 (20.5)	
≥61 years	293 (30.1)	229 (28.1)	64 (39.8)	
Residence				0.283
Provincial capital	282 (28.9)	233 (28.6)	49 (30.4)	
Satellite city	272 (27.9)	240 (29.5)	32 (19.9)	
Other city in province	120 (12.3)	104 (12.8)	16 (9.9)	
Outside province	301 (30.9)	237 (29.1)	64 (39.8)	

Values are median (interquartile range) or *n* (%). SMD denotes absolute standardized mean difference; for categorical variables with more than two levels, an omnibus SMD was calculated as the Mahalanobis distance between the category-proportion vectors of the two groups. The nonterminal clinical harm count is the number of the following present before the first complaint: ICU admission, reoperation, nursing-related friction, and invasive manipulation failure. Cost was counted from admission to the first complaint date, or over the whole admission for complaints first made after discharge.

**Table 2:** Final primary logistic model and point score.

Predictor	Beta Coefficient	Standard Error	Adjusted OR (95% CI)	*p* Value	Point Score
ICU admission before first complaint	0.673	0.194	1.96 (1.34–2.87)	<0.001	1
Invasive manipulation failure before first complaint	1.389	0.210	4.01 (2.66–6.05)	<0.001	2
Communication-context factor before first complaint	0.969	0.231	2.64 (1.68–4.14)	<0.001	1
Anger documented at the first complaint communication	1.240	0.199	3.46 (2.34–5.11)	<0.001	2
Intercept	−3.353	0.245	-	-	-

The outcome was high-grade dispute escalation, defined as event severity grades 7–10 versus grades 1–6. OR, odds ratio; CI, confidence interval; -, not applicable. Linear predictor (*LP*) = −3.353 + 0.673 × ICU admission + 1.389 × invasive manipulation failure + 0.969 × communication-context factor + 1.240 × anger at the first complaint, with each predictor coded 1 if present and 0 if absent; estimated probability = 1/[1 + exp(−*LP*)]. Point scores were obtained by dividing each coefficient by the smallest coefficient (0.673) and rounding to the nearest integer; they are simplified for bedside interpretability, and all performance estimates used the regression-based predicted probabilities.

## Data Availability

De-identified data and analysis code may be made available from the corresponding author upon reasonable request and subject to institutional ethics and data-use approvals.

## Supplementary Materials

The following supporting information can be downloaded at: https://www.mdpi.com/article/10.3390/healthcare14203360/s1, Figure S1: Prediction timepoint and information windows for the primary model; Figure S2: Adjusted associations in the primary logistic regression model; Figure S3: Observed high-grade event rates by model-defined risk stratum; Figure S4: Decision curve analysis of the primary early-warning model; Figure S5: Sensitivity analysis for alternative high-grade endpoint definitions; Table S1: Operational grouping of institutional event severity grades; Table S2: Event severity grade distribution, overall and by year; Table S3: Definitions of the components of the communication-context indicator; Table S4: Variable definitions and modeling roles; Table S5: Time-availability audit for candidate predictors; Table S6: Univariable logistic analyses; Table S7: Predictor selection in the training cohort and comparison of candidate models; Table S8: Machine-learning comparison in the test cohort; Table S9: Permutation importance in the test cohort; Table S10: Sensitivity of the communication variables to misclassification and omission; Table S11: Performance and calibration across patient groups; Table S12: Model performance, temporal validation, and operating characteristics at fixed thresholds; Table S13: Calibration-group summary; Table S14: Risk stratification by primary-model estimated probability; Table S15: Decision curve analysis values; Table S16: Sensitivity analyses; File S1: TRIPOD + AI checklist.
