# Supplementary material for: Risk-Informed Complaint Management in Neurosurgery: Development and Temporal Validation of an Early-Warning Model for Dispute Escalation
Source: Healthcare (Basel). 2026 Oct 9;14(20):3360. doi: 10.3390/healthcare14203360 (PMC13704672; doi:10.3390/healthcare14203360)
Supplement: Supplementary file 1 [file healthcare-14-03360-s001.zip › File_S1_TRIPOD-AI_checklist.pdf]

File S1. TRIPOD+AI checklist for "Risk-Informed Complaint Management in Neurosurgery: Development and Temporal Validation of an Early-Warning Model for Dispute Escalation"

Item descriptions are abbreviated; the full wording is given in Collins GS, et al. BMJ 2024;385:e078378. Locations refer to the revised manuscript.

Section	Item	Checklist item (abbreviated)	Where reported
Title	1	Study type, target population, and outcome	Title
Abstract	2	See TRIPOD+AI for Abstracts	Abstract
Introduction	3a	Health-care context and rationale	Section 1, paragraphs 1–3
	3b	Target population, intended purpose, and users	Section 1, paragraphs 4–6; Section 2.4
	3c	Known health inequalities between sociodemographic groups	Section 2.6 (support-needs profile); Section 4, limitations
	4	Objectives (development, evaluation, or both)	Section 1, final paragraph
Methods	5a	Data sources and their representativeness	Sections 2.1–2.2
	5b	Dates of data collection and follow-up	Sections 2.1 and 2.5; Section 3.1 (timing of escalation and final grading)
	6a	Setting, number and location of centres	Section 2.1 (single tertiary centre)
	6b	Eligibility criteria	Section 2.3; Figure 1
	6c	Treatments received and how handled	Not applicable (administrative outcome; clinical events before time zero are predictors, Section 2.6)
	7	Data preparation and quality checking	Sections 2.3 and 2.7
	8a	Outcome definition, time horizon, and assessment	Section 2.5; Table S1
	8b	Qualifications of outcome assessors (subjective outcome)	Section 2.5 (director of the complaint-management office; same adjudicator 2016–2025); reproducibility assessed by two re-graders familiar with the grading criteria in a stratified random sample of 150 events (Sections 2.5 and 3.1)
	8c	Blinding of outcome assessment	Section 2.5 (original grading not blinded to communication records; re-graders had access to the same records but were unaware of the recorded grade and study-coded predictor values)
	9a	Initial choice and pre-selection of predictors	Sections 2.6 and 2.9; Table S7
	9b	Definition and timing of predictors; blinding	Sections 2.6 and 2.7; Tables S3–S5
	9c	Qualifications of predictor assessors (subjective predictors)	Section 2.6 (study abstractor not blinded to the final grade; a second abstractor unaware of the final grade and original codes re-coded 150 events, with agreement in Section 3.1)
	10	Sample size	Section 2.3 (all eligible events used; no formal calculation); limitations in Section 4
	11	Missing data	Section 2.7; misclassification analysis in Section 2.14 and Table S10
	12a	Data partitioning for development and evaluation	Section 2.11 (temporal split by year of first complaint)

	12b	Handling of predictors	Section 2.6 (binary indicators)
	12c	Model type, building steps, and internal validation	Sections 2.9, 2.10, and 2.14
	12d	Heterogeneity across clusters	Not assessable (no patient or team identifiers); Section 2.2 and limitations
	12e	Performance measures and plots	Sections 2.10–2.13
	12f	Model updating or recalibration	Not performed; recalibration strategy in Section 4
	12g	How predictions were calculated in evaluation	Section 2.11 (training-cohort model applied unchanged)
	13	Class imbalance methods	No resampling; PR-AUC reported (Section 2.10)
	14	Approaches to fairness	Sections 2.6 and 2.14; Table S11
	15	Model output and thresholds	Sections 2.9, 2.11, and 2.12
	16	Differences between development and evaluation data	Section 2.11; Section 3.1 (event numbers by cohort)
	17	Ethics approval and consent	Section 2.16; back matter
Open science	18a	Funding	Funding statement
	18b	Conflicts of interest	Competing interests statement
	18c	Protocol	Protocol reviewed by the ethics committee; not publicly available
	18d	Registration	Not registered
	18e	Data sharing	Availability of data and materials statement
	18f	Code sharing	Availability of data and materials statement (not publicly available; on request from the corresponding author)
Patient and public involvement	19	Patient and public involvement	No patient or public involvement
Results	20a	Participant flow and outcome numbers	Section 3.1; Figure 1
	20b	Participant characteristics	Section 3.2; Table 1
	20c	Comparison of development and evaluation data	Section 3.1 (event rates by cohort); predictor distributions by cohort not reported
	21	Numbers of participants and events in each analysis	Section 3.1; Tables S12 and S16
	22	Full model specification	Table 2 with the linear predictor and intercept; Figure 2
	23a	Performance estimates with confidence intervals; subgroups	Section 3.4; Tables S11–S13 and S16
	23b	Heterogeneity across clusters	Not applicable (single centre)
	24	Model updating results	Not applicable
Discussion	25	Interpretation, including fairness	Section 4
	26	Limitations	Section 4, limitations paragraph
	27a	Handling of poor-quality or unavailable input data	Section 2.7 (undocumented features coded as absent)
	27b	User interaction and required expertise	Section 4, implementation paragraph
	27c	Next steps and generalizability	Section 4, final paragraphs; Section 5
