# Supplementary material for: Risk-Informed Complaint Management in Neurosurgery: Development and Temporal Validation of an Early-Warning Model for Dispute Escalation
Source: Healthcare (Basel). 2026 Oct 9;14(20):3360. doi: 10.3390/healthcare14203360 (PMC13704672; doi:10.3390/healthcare14203360)
Supplement: Supplementary file 1 [file healthcare-14-03360-s001.zip › Supplementary Materials revised.pdf]

Supplementary Figure Legends and Tables

Supplementary Figures

Figure S1. Prediction timepoint and information windows for the primary model.

Figure S2. Adjusted associations in the primary non-sociodemographic logistic regression model.

Figure S3. Observed high-grade event rates by model-defined risk stratum.

Figure S4. Decision curve analysis of the primary early-warning model.

Figure S5. Sensitivity analysis for alternative high-grade endpoint definitions.

Supplementary Tables

Table S1. Operational grouping of institutional event severity grades

Table S2. Event severity grade distribution, overall and by year

Table S3. Definitions of the components of the communication-context indicator

Table S4. Variable definitions and modeling roles

Table S5. Time-availability audit for candidate predictors

Table S6. Univariable logistic analyses for high-grade dispute escalation

Table S7. Predictor selection in the training cohort and comparison of candidate models

Table S8. Machine-learning comparison in the test cohort

Table S9. Permutation importance in the test cohort

Table S10. Sensitivity of the communication variables to misclassification and omission

Table S11. Performance and calibration across patient groups

Table S12. Model performance, temporal validation, and operating characteristics at fixed thresholds

Table S13. Calibration-group summary

Table S14. Risk stratification by primary-model estimated probability

Table S15. Decision curve analysis values

Table S16. Sensitivity analyses

File S1. TRIPOD+AI checklist

Supplementary Figures

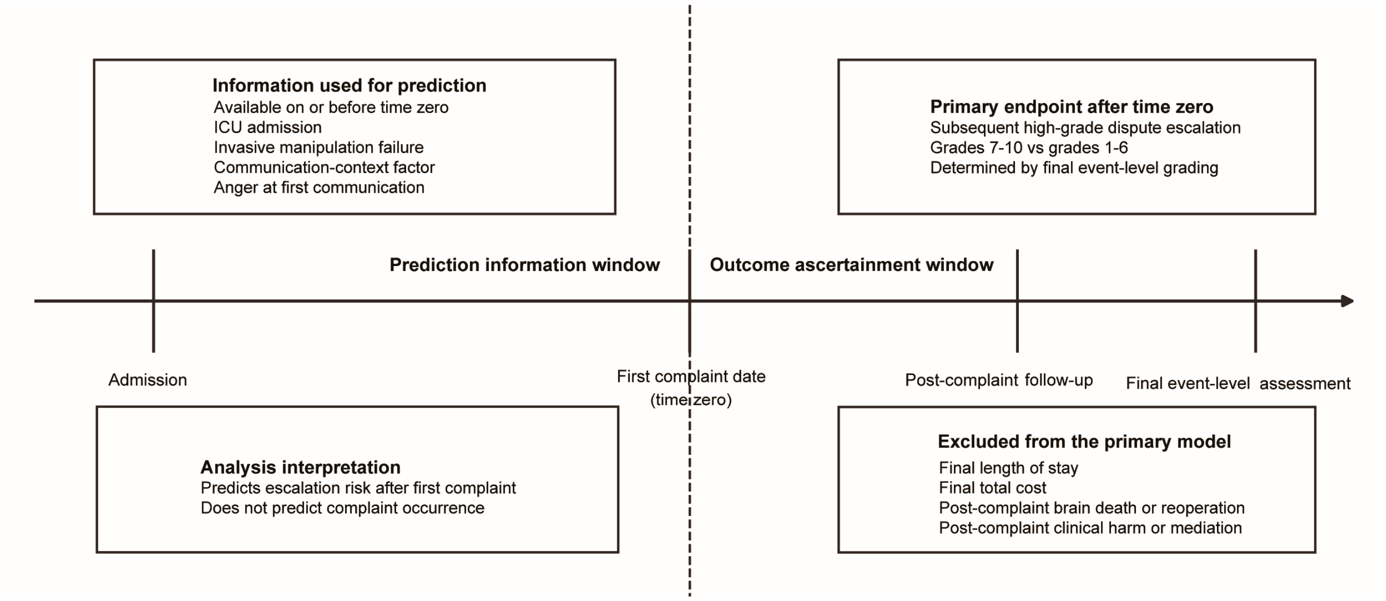

Figure S1. Prediction timepoint and information windows for the primary model.

Time zero was defined as the date of the first complaint. Eligible predictors for the primary model were restricted to clinical and communication variables recorded on or before time zero. The primary endpoint was subsequent escalation to a high-grade dispute after time zero. Post-complaint variables, including final length of stay, final total cost, post-complaint brain death or reoperation, and post-complaint clinical harm or mediation, were excluded from the primary model.

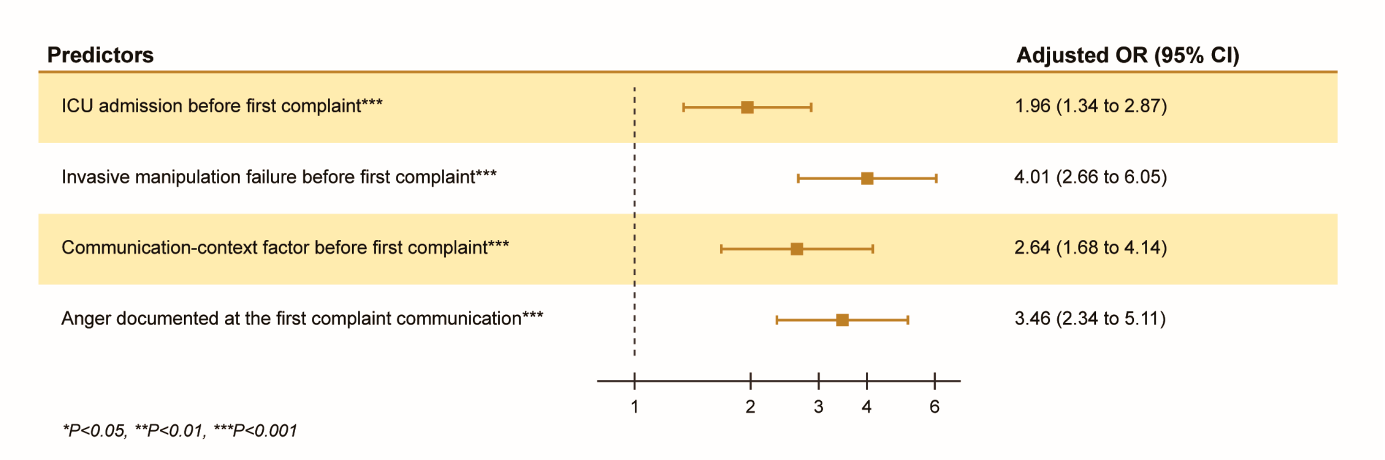

Figure S2. Adjusted associations in the primary non-sociodemographic logistic regression model.

Odds ratios are shown on a log scale with 95% confidence intervals. *P* values are from the primary multivariable logistic regression model.

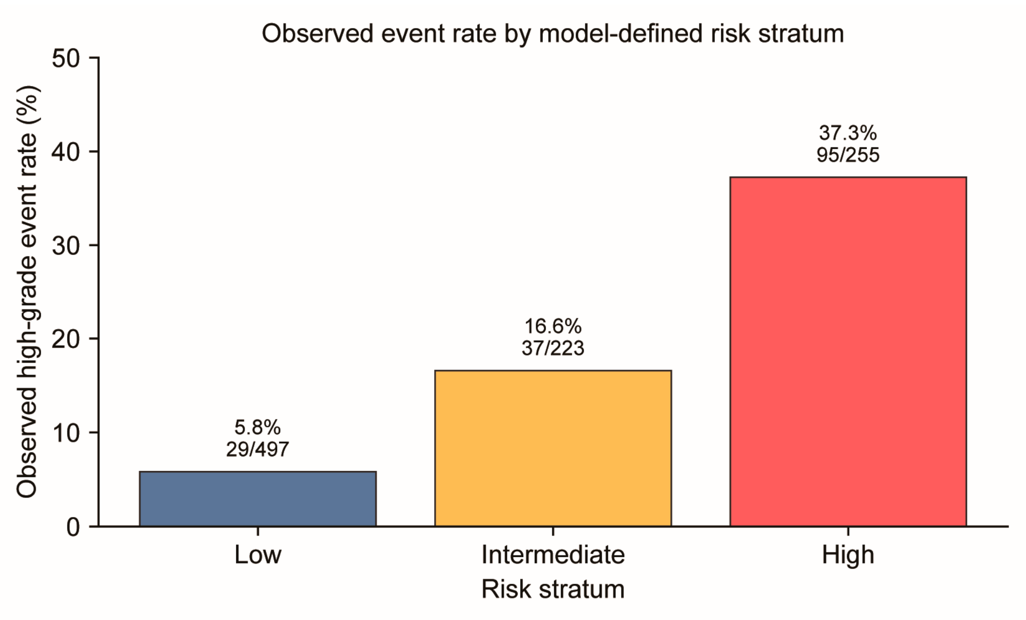

Figure S3. Observed high-grade event rates by model-defined risk stratum.

Risk strata were defined by primary-model predicted probability: low risk, <0.10; intermediate risk, 0.10 to <0.20; and high risk, ≥0.20.

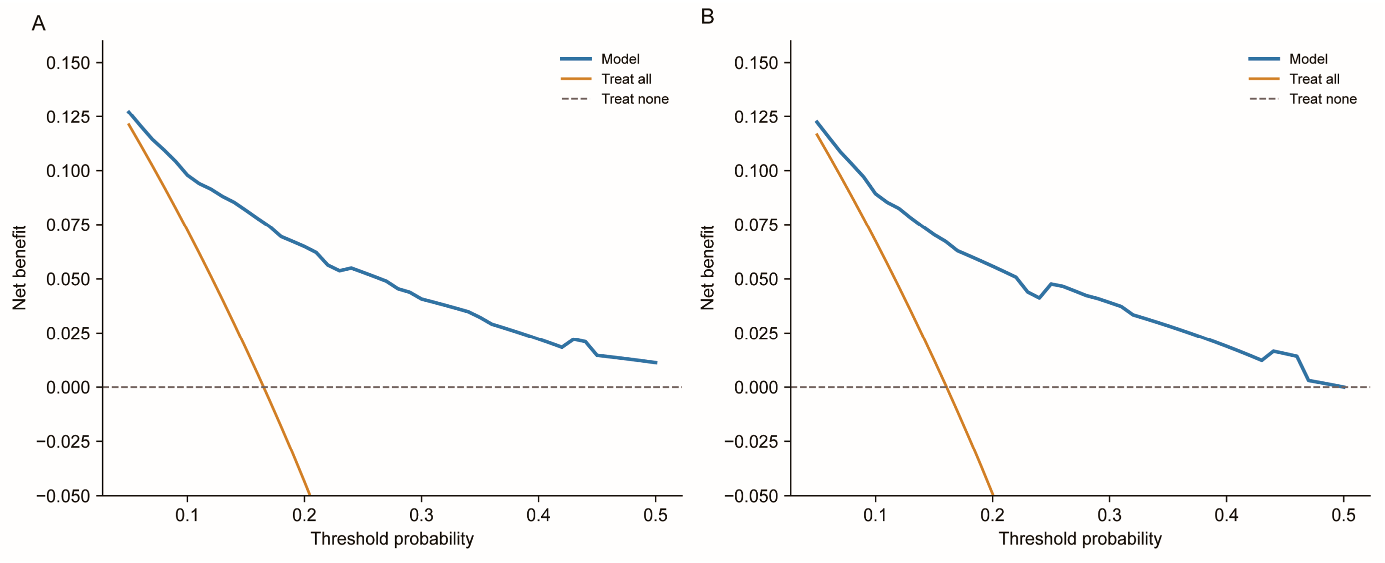

Figure S4. Decision curve analysis of the primary early-warning model.

Decision curve analysis showed the net benefit of the model across threshold probabilities in the full apparent cohort (**A**) and temporal test cohort (**B**), compared with treat-all and treat-none strategies.

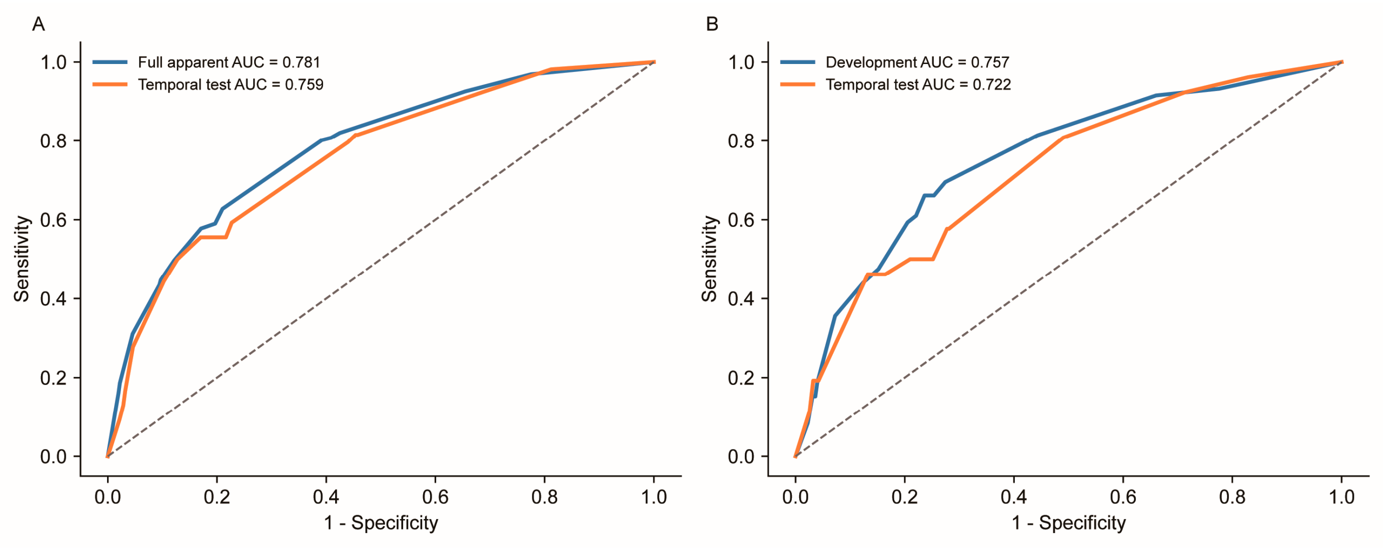

Figure S5. Sensitivity analysis for alternative high-grade endpoint definitions.

Receiver operating characteristic curves are shown for the primary endpoint definition (grades 7-10) and the stricter alternative endpoint definition (grades 8-10).

Supplementary Tables

Table S1. Operational grouping of institutional event severity grades

Grade	Endpoint class	Grade criterion	Required source record
1	Non-high-grade	1. Minor concern or dissatisfaction resolved by routine explanation at first contact.<br>2. No alleged clinical error requiring departmental review.<br>3. No written demand, compensation, mediation, or legal escalation.	1. Complaint log<br>2. Routine response note<br>3. Closure note
2	Non-high-grade	1. Formal complaint is registered.<br>2. Issue is limited to service, process, cost explanation, or communication concern.<br>3. Resolved by complaint office or staff written response without departmental meeting or escalation.	1. Formal complaint record<br>2. Written reply<br>3. Closure note
3	Non-high-grade	1. Low-severity complaint requiring departmental acknowledgement or written clarification.<br>2. No department-level review meeting is required.<br>3. No compensation negotiation, formal mediation, hospital-level intervention, or legal process.	1. Department acknowledgement<br>2. Written clarification<br>3. Closure note
4	Non-high-grade	1. Alleged care-process deficiency, repeated dissatisfaction, or responsible-team discussion requires department-level review.<br>2. Handling remains within the department.<br>3. No hospital-level intervention, formal mediation, settlement, or legal pathway.	1. Department review note<br>2. Meeting record<br>3. Handling decision
5	Non-high-grade	1. Persistent department-level dispute requiring enhanced communication or senior departmental involvement.<br>2. Mediation preparation may be documented, but formal mediation has not started.<br>3. No hospital-level decision, compensation decision,	1. Senior department note<br>2. Escalation note<br>3. Mediation-preparation record

Grade	Endpoint class	Grade criterion	Required source record
		or legal involvement.	
6	Non-high-grade	1. Unresolved or complex department-level complaint coordinated by the complaint office.<br>2. Highest non-high-grade level before formal high-grade escalation.<br>3. Closed without hospital-level adjudication, formal mediation, compensation decision, legal involvement, or administrative penalty.	1. Complaint-office coordination record<br>2. Department final handling record<br>3. Documentation showing no formal mediation, legal involvement, or administrative escalation
7	High-grade escalation	1. Hospital-level complaint-management intervention is required after the first complaint.<br>2. Examples include hospital-led multidisciplinary review, senior administrative meeting, or hospital-level written decision.<br>3. No formal mediation or legal pathway is required for this grade if those define higher grades.	1. Hospital meeting minutes<br>2. Hospital escalation log<br>3. Hospital-level written decision
8	High-grade escalation	1. Formal mediation or settlement pathway is initiated after the first complaint.<br>2. Examples include third-party mediation, documented compensation negotiation, settlement agreement, or hospital-level compensation decision.<br>3. The mediation record should be dated after time zero.	1. Mediation file<br>2. Settlement agreement<br>3. Compensation approval record
9	High-grade escalation	1. Legal, regulatory, police, or higher administrative pathway is initiated.<br>2. Examples include lawyer letter, court filing, regulatory complaint, police authority involvement, or administrative penalty process.<br>3. The external or higher-level pathway should be documented after the first complaint.	1. Legal, regulatory, police, or administrative file<br>2. Official correspondence<br>3. Record of external or higher administrative pathway

Grade	Endpoint class	Grade criterion	Required source record
10	High-grade escalation	1. Final high-consequence legal, regulatory, administrative, or institutional outcome is documented.<br>2. Examples include final legal decision, major settlement or compensation, formal institutional sanction, or crisis-level hospital handling.<br>3. The event has substantial institutional consequence and should not be described as objective legal severity unless independently adjudicated.	1. Final legal or administrative decision<br>2. Major settlement or compensation record<br>3. Sanction record or crisis-handling file

The institutional event severity system is an ordinal 10-grade scale: grade 1 indicates the lowest severity and grade 10 the highest. Higher grades indicate greater management and administrative consequences. Grades were assigned during routine complaint management, not for this study. Throughout 2016–2025, the final grade of each event was assigned by the director of the hospital complaint-management office, with input from the responsible clinical department when needed, and the criteria above were not revised. The director had access to the complete complaint file, including communication records. Each event was graded once in routine practice; agreement with two independent re-graders in a stratified random sample of 150 events is reported in Section 3.1 of the main text (Fleiss' κ, 0.928 for the 10-level grade and 0.958 for high-grade escalation). The retrospective prediction analysis used the recorded final grade and did not reassign grades. Grades 7–10 were grouped as high-grade escalation because they indicate hospital-level or higher dispute-management consequences.

Table S2. Event severity grade distribution, overall and by year

Event severity grade	Events, n	Events, %	Analysis group
1	87	8.9	Non-high-grade
2	116	11.9	Non-high-grade
3	171	17.5	Non-high-grade
4	180	18.5	Non-high-grade
5	167	17.1	Non-high-grade
6	93	9.5	Non-high-grade
7	76	7.8	High-grade
8	45	4.6	High-grade
9	29	3.0	High-grade
10	11	1.1	High-grade
Total	975	100.0	

The primary endpoint defined high-grade events as grades 7-10 and non-high-grade events as grades 1-6.

Table S2 (continued). High-grade events by year of first complaint

First complaint year	Events, n	High-grade events, n	High-grade events, %
2016	91	12	13.2
2017	76	10	13.2
2018	86	16	18.6
2019	112	17	15.2
2020	86	20	23.3
2021	88	13	14.8
2022	100	19	19.0

First complaint year	Events, n	High-grade events, n	High-grade events, %
2023	112	16	14.3
2024	113	24	21.2
2025	111	14	12.6
Trend			OR per year 1.01; $P = 0.69$

The trend was tested by logistic regression of high-grade escalation on calendar year. The grading criteria and the adjudicator were unchanged throughout 2016–2025.

Table S3. Definitions of the components of the communication-context indicator

Component documented on or before the first complaint	Coding definition
Multiple relatives involved	At least two different relatives directly took part in communication with clinical staff about diagnosis or treatment; relatives who were only mentioned were not counted.
Relatives' disagreement	The record explicitly described disagreement among relatives about treatment, prognosis, responsibility, or demands.
Lack of communication	The record explicitly described missing or delayed explanation of the diagnosis, treatment, risks, change in condition, or costs; dissatisfaction alone was not counted.
Open audio recording	The patient or relatives visibly recorded audio during communication or openly stated that they were recording; unconfirmed suspicion was not counted.
Open video recording	As for open audio recording, but for video recording.
Offensive language	The record explicitly quoted or described insulting language directed at staff; anger alone was not counted.
Depressed mood	The record explicitly described grief, crying, or withdrawal of the patient or relatives; no psychiatric diagnosis was inferred.
Reasonable doubt	The record documented a specific question about a clinical decision, outcome, or cost; this did not imply that the concern was medically substantiated.
Defensive attitude	The record explicitly described the complainant refusing explanation or further communication because of distrust; raising questions alone was not counted.

The communication-context factor was coded as present when any component was explicitly documented on or before the date of the first formal complaint, and as absent otherwise; anger at the first complaint was coded separately. Only the composite indicator was retained in the analytic dataset, so component-level frequencies and analyses are not available.

Table S4. Variable definitions and modeling roles

Construct	Operational definition	Modeling role
Primary endpoint	High-grade complaint escalation: grades 7-10 vs grades 1-6	Outcome
Prediction timepoint	First documented complaint/dispute signal	Time zero
ICU before complaint	ICU admission documented on or before time zero	Primary predictor
Invasive failure before complaint	Unsuccessful attempt at external ventricular drain, lumbar drain, central venous catheter placement, or lumbar puncture, with the procedure abandoned or repeated, documented on or before time zero; complications after a successful procedure and surgical procedures not included	Primary predictor
Communication-context factor before complaint	Any eligible pre-complaint communication-context factor, excluding anger	Primary predictor
Anger documented at the first complaint communication	Complainant described in the first-complaint record with expressions such as emotional agitation, strong dissatisfaction, or an on-site argument	Primary predictor
Sociodemographic support-needs profile	Primary school or below, freelance/unemployed occupation, age ≥61 years, or residence outside province	Descriptive and fairness analyses; not offered as a candidate predictor (stigmatization concern)
Reoperation before complaint	Reoperation documented on or before time zero	Candidate predictor

Nursing-related friction before complaint	Nursing-related friction documented on or before time zero	Candidate/descriptive predictor
Brain death before complaint	Brain death documented on or before time zero	Sensitivity predictor
Cost ≥45,000 CNY before complaint	Cumulative hospitalization cost ≥45,000 CNY from admission to time zero (whole admission for complaints first made after discharge)	Candidate predictor
Risk probability	Fitted probability from the primary logistic model	Risk stratification
Risk stratum	Low <0.10; intermediate 0.10 to <0.20; high ≥0.20	Risk stratification
Final cost and final stay	Final hospitalization cost and length of stay	Excluded to avoid look-ahead bias

The primary model used only clinical and communication-related information available on or before time zero. Final cost, final length of stay, and post-complaint events were excluded from the primary model to avoid look-ahead bias. Candidate predictors were selected by univariable screening ($P < 0.05$) followed by multivariable logistic regression ($P < 0.05$); see Table S7.

Table S5. Time-availability audit for candidate predictors

Variable	Model-ready records, n	Positive by time zero, n (%)	Time-zero role
First complaint date	975	-	Defines time zero
Final event severity grade	975	-	Outcome only
ICU admission before first complaint	975	455 (46.7)	Available by time zero
Reoperation before first complaint	975	121 (12.4)	Available by time zero
Nursing-related friction before first complaint	975	171 (17.5)	Available by time zero
Invasive manipulation failure before first complaint	975	157 (16.1)	Available by time zero
Brain death before first complaint	975	22 (2.3)	Sensitivity predictor
Communication-context factor before first complaint	975	602 (61.7)	Available by time zero
Anger documented at the first complaint communication	975	197 (20.2)	Available by time zero
Cost ≥45,000 CNY before first complaint	975	524 (53.7)	Candidate
Sociodemographic support-needs profile	975	784 (80.4)	Descriptive only
Risk probability	975	-	Model output
Temporal cohort label	975	-	Validation split

Variables labelled as available by time zero were coded only from information documented on or before the first complaint date. Outcome variables and model outputs were not candidate predictors.

Table S6. Univariable logistic analyses for high-grade dispute escalation

Variable	Comparison or unit	OR (95% CI)	*P* value
Days from admission to first complaint	Per 7-day increase	0.92 (0.71-1.20)	0.534
Nonterminal clinical harm count before complaint	Per 1-factor increase	2.18 (1.80-2.62)	<0.001
First complaint during hospitalization	Present vs absent	0.92 (0.66-1.30)	0.646
ICU admission before first complaint	Present vs absent	2.49 (1.75-3.54)	<0.001
Reoperation before first complaint	Present vs absent	2.13 (1.37-3.31)	<0.001
Nursing-related friction before first complaint	Present vs absent	1.58 (1.05-2.38)	0.028
Invasive manipulation failure before first complaint	Present vs absent	4.92 (3.36-7.22)	<0.001
Brain death before first complaint	Present vs absent	0.50 (0.12-2.16)	0.352
Communication-context factor before first complaint	Present vs absent	3.33 (2.18-5.10)	<0.001
Anger documented at the first complaint communication	Present vs absent	4.62 (3.21-6.65)	<0.001
Cost ≥45,000 CNY before first complaint	Present vs absent	1.22 (0.86-1.71)	0.263
Sociodemographic support-needs profile	Present vs absent	3.19 (1.76-5.75)	<0.001
Female vs male	Category contrast	0.88 (0.62-1.23)	0.442
Middle/high school vs college or above	Category contrast	0.88 (0.58-1.32)	0.524
Primary school or below vs college or above	Category contrast	0.94 (0.62-1.43)	0.765
Self-employed vs employee/public institution or retired	Category contrast	0.75 (0.49-1.14)	0.173
Freelance/unemployed vs employee/public institution or retired	Category contrast	0.93 (0.62-1.38)	0.709

11-39 years vs 0-10 years	Category contrast	1.68 (0.86-3.26)	0.128
40-60 years vs 0-10 years	Category contrast	1.63 (0.81-3.29)	0.171
≥61 years vs 0-10 years	Category contrast	2.59 (1.34-4.99)	0.005
Satellite city vs provincial capital	Category contrast	0.63 (0.39-1.03)	0.063
Other city in province vs provincial capital	Category contrast	0.73 (0.40-1.35)	0.315
Outside province vs provincial capital	Category contrast	1.28 (0.85-1.94)	0.236

Each row reports a separate univariable logistic regression in the full cohort with high-grade institutional dispute-management escalation, defined as grades 7–10, as the outcome. Univariable results restricted to the 2016–2022 training cohort are given in Table S7.

Table S7. Predictor selection in the training cohort and comparison of candidate models

A. Univariable screening and multivariable step in the full and training cohorts

**Candidate**	**Full cohort, univariable OR; P**	**Full cohort, multivariable OR; P**	**Training cohort, univariable OR; P**	**Training cohort, multivariable OR; P**	**Retained**
ICU admission	2.49; <0.001	1.91; 0.001	2.51; <0.001	1.93; 0.006	Yes
Reoperation	2.13; <0.001	1.39; 0.200	2.56; <0.001	1.53; 0.175	No
Nursing-related friction	1.58; 0.028	1.26; 0.322	1.33; 0.258	not entered	No
Invasive manipulation failure	4.92; <0.001	3.89; <0.001	5.82; <0.001	4.44; <0.001	Yes
Communication-context factor	3.33; <0.001	2.61; <0.001	3.44; <0.001	2.52; 0.001	Yes
Anger at the first complaint	4.62; <0.001	3.37; <0.001	4.75; <0.001	3.68; <0.001	Yes
Cost ≥45,000 CNY	1.22; 0.263	not entered	1.28; 0.252	not entered	No

B. Candidate models ranked by training-cohort AIC (all 127 combinations of the seven candidates)

**Predictors (training-cohort fit)**	**AIC**	**AIC rank**	**Training AUC**	**Test AUC**	**Test PR-AUC**	**Test Brier**	**Test calibration slope**
**ICU admission + Invasive manipulation failure + Communication-context factor + Anger at first complaint**	**479.1**	**1**	**0.792**	**0.759**	**0.364**	**0.119**	**0.839**
ICU admission + Reoperation + Invasive	479.4	2	0.791	0.759	0.379	0.120	0.840

Predictors (training-cohort fit)	AIC	AIC rank	Training AUC	Test AUC	Test PR-AUC	Test Brier	Test calibration slope
manipulation failure + Communication-context factor + Anger at first complaint							
ICU admission + Invasive manipulation failure + Communication-context factor + Anger at first complaint + Cost ≥45,000 CNY	481.1	3	0.790	0.758	0.370	0.119	0.837
ICU admission + Nursing-related friction + Invasive manipulation failure + Communication-context factor + Anger at first complaint	481.1	4	0.792	0.762	0.372	0.119	0.842
ICU admission + Reoperation + Invasive manipulation failure + Communication-context factor + Anger at first complaint + Cost ≥45,000 CNY	481.3	5	0.791	0.760	0.385	0.120	0.835
ICU admission + Reoperation + Nursing-related friction + Invasive manipulation failure + Communication-context factor + Anger at first complaint	481.4	6	0.791	0.763	0.385	0.120	0.841
ICU admission +	483.1	7	0.790	0.759	0.375	0.119	0.839

Predictors (training-cohort fit)	AIC	AIC rank	Training AUC	Test AUC	Test PR-AUC	Test Brier	Test calibration slope
Nursing-related friction + Invasive manipulation failure + Communication-context factor + Anger at first complaint + Cost ≥45,000 CNY							
ICU admission + Reoperation + Nursing-related friction + Invasive manipulation failure + Communication-context factor + Anger at first complaint + Cost ≥45,000 CNY	483.3	8	0.791	0.761	0.387	0.120	0.837
Reoperation + Invasive manipulation failure + Communication-context factor + Anger at first complaint	485.0	9	0.776	0.741	0.366	0.120	0.839
Invasive manipulation failure + Communication-context factor + Anger at first complaint	485.4	10	0.774	0.739	0.338	0.120	0.832
Nursing-related friction + Invasive manipulation failure + Communication-context factor + Anger at first complaint	487.3	13	0.775	0.748	0.352	0.120	0.837
Invasive manipulation failure +	487.4	14	0.769	0.738	0.346	0.120	0.832

Predictors (training-cohort fit)	AIC	AIC rank	Training AUC	Test AUC	Test PR-AUC	Test Brier	Test calibration slope
Communication-context factor + Anger at first complaint + Cost ≥45,000 CNY							
ICU admission + Reoperation + Invasive manipulation failure + Anger at first complaint	489.2	17	0.768	0.733	0.338	0.123	0.777

C. Selection frequency when the full selection procedure was repeated in 1000 bootstrap samples

Candidate	Selected in bootstrap samples, %
ICU admission	92.4
Reoperation	27.4
Nursing-related friction	17.2
Invasive manipulation failure	100.0
Communication-context factor	99.3
Anger at the first complaint	100.0
Cost ≥45,000 CNY	2.1
Exact four-predictor set (ICU, invasive failure, communication context, anger)	53.5

Selection rule: univariable $P < 0.05$, then removal of candidates with multivariable $P \geq 0.05$. The same four predictors were selected in the full cohort, in the training cohort alone, and with thresholds of 0.10 at both steps. Panel B lists the ten models with the lowest training-cohort AIC and the best alternative four-predictor models; the selected model is shown in bold. Test metrics refer to the 2023–2025 temporal test cohort. With the selection procedure repeated in each bootstrap sample, the optimism-corrected AUC was 0.774 (apparent AUC 0.781).

Table S8. Machine-learning comparison in the test cohort

Model	Test, n	Test events	AUC	PR-AUC	Brier score
Primary logistic regression	336	54	0.759	0.364	0.119
LASSO logistic regression	336	54	0.759	0.364	0.119
Elastic-net logistic regression	336	54	0.759	0.364	0.119
Random forest	336	54	0.757	0.368	0.118
Gradient boosting	336	54	0.759	0.364	0.119

Models were trained in the 2016-2022 cohort and evaluated in the 2023-2025 test cohort, defined by first complaint year.

Table S9. Permutation importance in the test cohort

Feature	Mean AUC decrease	SD
ICU admission before first complaint	0.022	0.013
Invasive manipulation failure before first complaint	0.056	0.019
Communication-context factor before first complaint	0.050	0.021
Anger documented at the first complaint communication	0.069	0.021

Permutation importance was calculated as the mean decrease in test cohort AUC.

Table S10. Sensitivity of the communication variables to misclassification and omission

A. Misclassification scenarios (200 simulated datasets per row)

Variable perturbed	Scenario	Proportion reclassified	Adjusted OR, median (2.5th–97.5th percentile)	Temporal AUC, median
Anger at first complaint	Random under-recording, any outcome (absent → present)	10%	2.67 (2.27–3.20)	0.743
Anger at first complaint	Random under-recording, any outcome (absent → present)	20%	2.27 (1.86–2.93)	0.738
Anger at first complaint	Under-recording in events that did not escalate (absent → present)	10%	2.14 (2.02–2.27)	0.731
Anger at first complaint	Under-recording in events that did not escalate (absent → present)	20%	1.46 (1.35–1.57)	0.720
Anger at first complaint	Over-recording in high-grade events (present → absent)	10%	2.88 (2.78–2.98)	0.748
Anger at first complaint	Over-recording in high-grade events (present → absent)	20%	2.33 (2.21–2.44)	0.735
Communication-context factor	Random under-recording, any outcome (absent → present)	10%	2.51 (2.18–2.96)	0.754
Communication-context factor	Random under-recording, any outcome (absent → present)	20%	2.48 (1.89–3.20)	0.751
Communication-context factor	Under-recording in events that did not escalate (absent → present)	10%	2.24 (2.14–2.33)	0.751

Variable perturbed	Scenario	Proportion reclassified	Adjusted OR, median (2.5th–97.5th percentile)	Temporal AUC, median
Communication-context factor	Under-recording in events that did not escalate (absent → present)	20%	1.87 (1.75–1.97)	0.746
Communication-context factor	Over-recording in high-grade events (present → absent)	10%	1.65 (1.56–1.74)	0.747
Communication-context factor	Over-recording in high-grade events (present → absent)	20%	1.12 (1.05–1.21)	0.733

B. Models omitting the communication variables

Model (fitted 2016–2022, tested 2023–2025)	AUC	PR-AUC	Brier score	Calibration intercept	Calibration slope
Primary model (all four predictors)	0.759	0.364	0.119	-0.132	0.839
Without communication-context factor	0.730	0.321	0.123	-0.119	0.782
Without anger at the first complaint	0.712	0.317	0.122	-0.142	0.946
ICU admission and invasive manipulation failure only	0.682	0.257	0.129	-0.139	0.782

Reference values in the primary model: adjusted OR 3.46 for anger and 2.64 for the communication-context factor; temporal AUC 0.759. In each simulated dataset, the stated proportion of eligible events was reclassified at random, the model was refitted in the full cohort (adjusted OR) and in the training cohort (temporal AUC). The scenarios represent undocumented features coded as absent and selective recording of the communication variables in complaints perceived as difficult.

Table S11. Performance and calibration across patient groups

A. Observed and predicted event rates by group

Cohort	Variable	Group	Events, n	High-grade, n	Observed, %	Mean predicted, %	O/E	AUC
Temporal test	Age group	0-10 years	37	1	2.7	17.2	0.16	0.625
Temporal test	Age group	11-39 years	125	22	17.6	16.9	1.04	0.805
Temporal test	Age group	40-60 years	76	11	14.5	16.5	0.88	0.696
Temporal test	Age group	≥61 years	98	20	20.4	19.4	1.05	0.754
Temporal test	Place of residence	Provincial capital	88	14	15.9	16.4	0.97	0.776
Temporal test	Place of residence	Satellite city	99	13	13.1	18.2	0.72	0.786
Temporal test	Place of residence	Other city in province	43	7	16.3	15.6	1.05	0.645
Temporal test	Place of residence	Outside province	106	20	18.9	18.8	1.00	0.771
Temporal test	Support-needs profile	Support-needs profile absent	71	4	5.6	14.9	0.38	0.728
Temporal test	Support-needs profile	Support-needs profile present	265	50	18.9	18.3	1.03	0.757
Temporal test	Timing of first complaint	First complaint during hospitalization	192	30	15.6	17.6	0.89	0.691
Temporal	Timing of first	First complaint	144	24	16.7	17.6	0.95	0.842

**Cohort**	**Variable**	**Group**	**Events, n**	**High-grade, n**	**Observed, %**	**Mean predicted, %**	**O/E**	**AUC**
test	complaint	after discharge						
Full apparent	Age group	0-10 years	123	12	9.8	17.4	0.56	0.821
Full apparent	Age group	11-39 years	339	52	15.3	15.9	0.96	0.821
Full apparent	Age group	40-60 years	220	33	15.0	16.0	0.94	0.692
Full apparent	Age group	≥61 years	293	64	21.8	17.3	1.27	0.791
Full apparent	Place of residence	Provincial capital	282	49	17.4	15.5	1.12	0.802
Full apparent	Place of residence	Satellite city	272	32	11.8	17.5	0.67	0.750
Full apparent	Place of residence	Other city in province	120	16	13.3	13.4	0.99	0.748
Full apparent	Place of residence	Outside province	301	64	21.3	17.7	1.20	0.799
Full apparent	Support-needs profile	Support-needs profile absent	191	13	6.8	14.0	0.49	0.715
Full apparent	Support-needs profile	Support-needs profile present	784	148	18.9	17.1	1.10	0.786
Full apparent	Timing of first complaint	First complaint during hospitalization	573	92	16.1	16.8	0.95	0.762
Full	Timing of first	First complaint	402	69	17.2	16.1	1.07	0.807

Cohort	Variable	Group	Events, n	High-grade, n	Observed, %	Mean predicted, %	O/E	AUC
apparent	complaint	after discharge						

B. Performance forgone by excluding the support-needs profile

Model	Support-needs OR (P)	Apparent AUC	Temporal AUC	Temporal PR-AUC	O/E, profile absent	O/E, profile present
Primary model	not included	0.781	0.759	0.364	0.38	1.03
Primary model + support-needs profile	2.83 (0.001)	0.798	0.778	0.383	0.68	0.95

O/E, ratio of the observed to the mean predicted high-grade event rate; values below 1 indicate overestimation. Temporal test cohort values use the model fitted in 2016–2022; full-cohort values are apparent. AUCs in small groups are imprecise, and the 0–10 years group of the test cohort contained a single high-grade event. The support-needs profile was not offered as a candidate predictor because of stigmatization concerns.

Table S12. Model performance, temporal validation, and operating characteristics at fixed thresholds

Metric	Estimate	95% CI	TP	FP	TN	FN	Additional information
Apparent AUC	0.781	0.741-0.818					Full analytic cohort; bootstrap 95% CI; resamples=1000
Optimism-corrected AUC	0.774	-					Bootstrap optimism correction with the complete selection procedure repeated; resamples=1000
Apparent PR-AUC	0.417	-					Full analytic cohort; average precision
Apparent Brier score	0.115	-					Full analytic cohort; lower is better
Apparent Youden threshold	0.192	-					Threshold from full apparent cohort primary model
Apparent sensitivity	0.627	0.550-0.698	101	171	643	60	Wilson 95% CI
Apparent specificity	0.790	0.761-0.817	101	171	643	60	Wilson 95% CI
Apparent PPV	0.371	0.316-0.430	101	171	643	60	Wilson 95% CI
Apparent NPV	0.915	0.892-0.933	101	171	643	60	Wilson 95% CI
Temporal validation AUC	0.759	0.687–0.826					Train 2016-2022; test 2023-2025
Temporal validation PR-AUC	0.364	0.263–0.496					Train 2016-2022; test 2023-2025; average precision
Temporal validation Brier score	0.119	0.096–0.144					Train 2016-2022; test 2023-2025; lower is better
Training-derived threshold	0.209	-					Youden threshold from 2016-2022 training set, applied to 2023-2025 test set
Temporal validation sensitivity	0.593	0.460-0.713	32	64	218	22	Wilson 95% CI

Temporal validation specificity	0.773	0.721-0.818	32	64	218	22	Wilson 95% CI
Temporal validation PPV	0.333	0.247-0.432	32	64	218	22	Wilson 95% CI
Temporal validation NPV	0.908	0.865-0.939	32	64	218	22	Wilson 95% CI
Temporal validation calibration intercept	-0.132	-0.500 to 0.203					Test set calibration-in-the-large
Temporal validation calibration slope	0.839	0.579–1.158					Test set logistic recalibration

AUC, area under the receiver-operating characteristic curve; PR-AUC, area under the precision-recall curve; CI, confidence interval; TP, true positives; FP, false positives; TN, true negatives; FN, false negatives; PPV, positive predictive value; NPV, negative predictive value. Temporal AUC, PR-AUC, Brier score, calibration intercept, and slope CIs are percentile intervals from 2000 bootstrap resamples of the test cohort; Wilson 95% CIs are shown for sensitivity, specificity, PPV, and NPV.

Table S12 (continued). Operating characteristics in the temporal test cohort at fixed thresholds

Threshold	TP	FP	TN	FN	Flagged, n (%)	Sensitivity (95% CI)	Specificity (95% CI)	PPV (95% CI)	NPV (95% CI)
0.10	44	129	153	10	173 (51.5%)	0.815 (0.692–0.896)	0.543 (0.484–0.600)	0.254 (0.195–0.324)	0.939 (0.891–0.966)
0.15	32	64	218	22	96 (28.6%)	0.593 (0.460–0.713)	0.773 (0.721–0.818)	0.333 (0.247–0.432)	0.908 (0.865–0.939)
0.20	32	64	218	22	96 (28.6%)	0.593 (0.460–0.713)	0.773 (0.721–0.818)	0.333 (0.247–0.432)	0.908 (0.865–0.939)

The four binary predictors yield 15 distinct predicted probabilities in the test cohort, none of them between 0.149 and 0.209. Thresholds of 0.15 and 0.20 therefore classify the test cohort identically to the training-derived Youden threshold of 0.209.

Table S13. Calibration-group summary

Dataset	Bin	Events, n	High-grade events, n	Mean predicted risk	Observed event rate
Full apparent cohort	Decile 1	98	3	0.034	0.031
Full apparent cohort	Decile 2	97	3	0.036	0.031
Full apparent cohort	Decile 3	98	6	0.064	0.061
Full apparent cohort	Decile 4	97	9	0.084	0.093
Full apparent cohort	Decile 5	98	8	0.084	0.082
Full apparent cohort	Decile 6	97	12	0.135	0.124
Full apparent cohort	Decile 7	97	17	0.153	0.175
Full apparent cohort	Decile 8	98	16	0.208	0.163
Full apparent cohort	Decile 9	97	33	0.325	0.340
Full apparent cohort	Decile 10	98	54	0.526	0.551
Temporal test cohort	Quintile 1	68	2	0.040	0.029
Temporal test cohort	Quintile 2	67	6	0.075	0.090

Temporal test cohort	Quintile 3	67	9	0.119	0.134
Temporal test cohort	Quintile 4	67	8	0.188	0.119
Temporal test cohort	Quintile 5	67	29	0.458	0.433

Full apparent cohort calibration groups use 10 equal-count groups. Temporal test cohort calibration groups use 5 equal-count groups because of the smaller test cohort.

Table S14. Risk stratification by primary-model estimated probability

Dataset	Risk stratum	Events, n	High-grade events, n	Observed high-grade event rate	Mean predicted risk
Full analytic cohort	Low (<0.10)	497	29	5.8%	6.1%
Full analytic cohort	Intermediate (0.10 to <0.20)	223	37	16.6%	15.1%
Full analytic cohort	High (≥0.20)	255	95	37.3%	38.1%
Temporal test cohort	Low (<0.10)	163	10	6.1%	6.2%
Temporal test cohort	Intermediate (0.10 to <0.20)	77	12	15.6%	14.7%
Temporal test cohort	High (≥0.20)	96	32	33.3%	39.2%

Risk strata were defined using estimated probability thresholds of <0.10, 0.10 to <0.20, and ≥0.20. Full analytic cohort strata used the final primary model estimated probabilities; temporal test cohort strata used probabilities from the model trained in 2016-2022 and applied to the 2023-2025 test cohort.

Table S15. Decision curve analysis values

Dataset	Threshold probability	Model net benefit	Treat-all net benefit
Full apparent cohort	0.05	0.126	0.121
Full apparent cohort	0.10	0.096	0.072
Full apparent cohort	0.15	0.075	0.018
Full apparent cohort	0.20	0.056	-0.044
Full apparent cohort	0.25	0.048	-0.113
Full apparent cohort	0.30	0.039	-0.193
Full apparent cohort	0.35	0.029	-0.284
Full apparent cohort	0.40	0.026	-0.391
Full apparent cohort	0.45	0.016	-0.518
Full apparent cohort	0.50	0.010	-0.670
Temporal test cohort	0.05	0.122	0.117
Temporal test cohort	0.10	0.088	0.067
Temporal test cohort	0.15	0.062	0.013
Temporal test cohort	0.20	0.048	-0.049
Temporal test cohort	0.25	0.042	-0.119
Temporal test cohort	0.30	0.034	-0.199
Temporal test cohort	0.35	0.025	-0.291
Temporal test cohort	0.40	0.014	-0.399
Temporal test cohort	0.45	0.005	-0.526
Temporal test cohort	0.50	0.000	-0.679

Net benefit was calculated across threshold probabilities from 0.05 to 0.50. Treat-none net benefit is zero by definition.

Table S16. Sensitivity analyses

Analysis	High-grade events, n	AUC	PR-AUC	Brier score
Add terminal clinical marker	54	0.761	0.365	0.119
Stricter endpoint, development cohort	59	0.757	0.233	0.077
Stricter endpoint, temporal test cohort	26	0.722	0.183	0.068
First complaints during hospitalization, refitted within subgroup	30	0.687	0.262	0.129
First complaints during hospitalization, primary model applied	30	0.691	0.273	0.131
First complaints after discharge, primary model applied	24	0.842	0.534	0.103

Brain death before first complaint was evaluated only as a sensitivity predictor because it is a severe terminal clinical marker. The grade 8–10 endpoint used a stricter high-grade definition; development and temporal-test results are listed separately. Subgroup rows refer to the 2023–2025 temporal test cohort.
